# Supplementary material for: Effects of changes in physical and sedentary behaviors on mental health and life satisfaction during the COVID-19 pandemic: Evidence from China
Source: PLoS One. 2022 Jun 21;17(6):e0269237. doi: 10.1371/journal.pone.0269237 (PMC9212146; doi:10.1371/journal.pone.0269237)
Supplement: Attachment — Submitted filename: Edited PONE-D-21-27357_R1.pdf [file pone.0269237.s002.pdf]

PLOS ONE

Effects of changes in physical and sedentary behaviors on mental health and life satisfaction during the COVID-19 pandemic: Evidence from China

--Manuscript Draft--

**Manuscript Number:**	PONE-D-21-27357R1
**Article Type:**	Research Article
**Full Title:**	Effects of changes in physical and sedentary behaviors on mental health and life satisfaction during the COVID-19 pandemic: Evidence from China
**Short Title:**	Changes in physical and sedentary behaviors and mental wellbeing during the COVID-19 pandemic
**Corresponding Author:**	Xi Chen<br>The Chinese University of Hong Kong<br>Hong Kong, HONG KONG
**Keywords:**	physical activity, sedentary behavior, mental health, life satisfaction, COVID-19 pandemic, China
**Abstract:**	Background: While restriction measures are critical in containing the COVID-19 outbreak, limited studies have investigated the behavioral and psychological impact of these measures. This study aimed to investigate the effects of physical and sedentary behavioral changes and online behavior during the COVID-19 pandemic on mental health and life satisfaction among the Chinese population.<br>Methods: The data were obtained from a cross-sectional survey of 2145 residents aged between 18 and 80 in Hubei province, China between March 23, 2020, and April 9, 2020.<br>Results: Participants who had high frequencies of physical activities before or during the COVID-19 outbreak exhibited higher levels of life satisfaction. Participants who increased their sitting time during the pandemic or kept sitting for more than eight hours before and during the pandemic reported worse mental health than those who maintained less sedentary behavior. Besides, participants who used the Internet for information seeking, communication, and entertainment more frequently reported better mental health and life satisfaction. In contrast, there was a positive association between commercial use of the Internet and symptoms of mental disorders<br>Conclusion: Given the link between physical and sedentary behavioral changes with worse mental wellbeing, strategies to reduce sedentariness and increase physical activity during the COVID-19 pandemic are needed.
**Order of Authors:**	Xi Chen
	Haiyan Gao
	Binbin Shu
	Yuchun Zou
**Opposed Reviewers:**	
**Response to Reviewers:**	Response Letter<br><br>Editor's Comment<br><br>1.Please clarify the reasons for the restrictions on data availability. Stating "data available upon reasonable request from the corresponding author" is not sufficient. Please explain your exceptional situation.<br><br>Response: We have clarified the reasons for the restrictions on data availability, as shown below.<br><br>"Data cannot be shared publicly because of data protection regulation. Data are available from the Chinese Social Quality Data Archive for researchers who meet the criteria for access to confidential data. The data are available for research upon

Powered by Editorial Manager® and ProduXion Manager® from Aries Systems Corporation

reasonable request and with permission from the Chinese Social Quality Data Archive: http://csqr.cass.cn/index.jsp"

2. This is a cross-sectional study using survey methodology. Your limitations section (pages 13-14) does a very good job of conveying this material about the cross-sectional nature of the design. As you note, this type of design does not allow statements about one variable causing a change in another variable. For this reason, statements such as "the commercial use of the internet seemed to increase mental disorders" (in the abstract) should be phrased as "there was an association between commercial use of the internet and symptoms of mental disorders." (Note comment 4 below that mental disorders per se were not assessed; rather, a self-report measure of symptoms of mental disorders was completed). Likewise, the statement "we hypothesized that (1) the lockdown may cause a decrease in PA and an increase in sedentary behavior" (page 6, as numbered) is not appropriate for this design. Please rewrite as "….the lockdown may be associated with changes in PA and sedentary behavior." More generally, throughout the writing, please edit to make sure the language used reflects the cross-sectional design and does not suggest causality or a longitudinal design.

Response: Thanks for your comment. We have revised the manuscript accordingly, using language that reflects the cross-sectional design. We detailed the changes below.

(1) We have rephrased the sentence "the commercial use of the internet seemed to increase mental disorders" (in the Abstract) as "there was a positive association between commercial use of the internet and symptoms of mental disorders."

(2) As the editor suggested, we have revised the first hypothesis as "the COVID-19 pandemic may be associated with changes in PA and sedentary behavior." (p.6)

(3) We have revised the second hypothesis, "a decrease in PA may deteriorate mental health and life satisfaction," as "a decrease in PA may be negatively associated with mental health and life satisfaction." (p.6)

3. Please clarify the consent process for the participants who went through a phone survey. Did participants who answered the questions via phone give oral consent, and those who did the online survey provide written consent?

Response: We have added information about the consent process in the revised manuscript (p.7).

"Participants who did the online survey provided written consent. Participants who answered the questions via phone gave oral consent, and the interviewer signed on a form pledging that he/she had gone through the proper procedures to obtain the verbal informed consent of the participant."

4. The self-report measure used does not permit a true diagnosis of mental disorders. I recommend using language that indicates that the measure reports on symptoms consistent with a potential mental disorder.

Response: Thanks for the comment. We have revised the manuscript using language that indicates that the measure reports on symptoms consistent with a potential mental disorder. For example,

(1) We rewrote the sentence "Mental health was measured by the 12-item Chinese Health Questionnaire scale (CHQ-12)" as "Symptoms of mental disorders were measured by the 12-item Chinese Health Questionnaire scale (CHQ-12)". (p.7)

(2) We have rephrased the sentence, "About 32.07% of the participants scored greater than or equal to 14 and could be regarded as having potential mental disorders." as "About 32.07% of the participants scored greater than or equal to 14 and could be regarded as having symptoms consistent with a potential mental disorder." (p.8)

(3) We have rephrased the sentence, "we fit multiple logistic regression models to

Powered by Editorial Manager® and ProduXion Manager® from Aries Systems Corporation

study the associations between changes in sitting time and PA during the lockdown period of COVID-19 and online behavior with potential mental disorders." as "we fit multiple logistic regression models to study the associations between changes in sitting time and PA during the lockdown period of COVID-19 and online behavior with symptoms of potential mental disorders."

5. What is meant by depression, page 10 as numbered? I don't see a measure of depression. Is depression used as a synonym for "mental disorders"? Please harmonize the language. If the self-report measure is a measure of mental disorders, then the term mental disorders should be used to refer to findings from this measure.

Response: Thanks for the comment. We used depression as a synonym for mental disorders in the original manuscript. We have harmonized the language in the revised manuscript, particularly in the results and discussion parts. For example, we rewrote the sentence, "kept sitting for more than 8 hours a day at both time points were more likely to develop depressive symptoms" as "kept sitting for more than 8 hours a day at both time points were more likely to experience symptoms of mental disorders" (p.1q).

We rewrote the sentence, "However, changes in the frequency of PA were not associated with depression" as "However, changes in the frequency of PA were not associated with symptoms of mental disorders" (p.11). Besides, we revised the sentence, "Previous studies also found that online shopping may be a negative coping strategy for stress and was associated with increased depression" as "Previous studies also found that online shopping may be a negative coping strategy for stress and was associated with increased symptoms of mental disorders." (p.13)

6. Please make sure all the dates and details given in the text about the pandemic are correct. It is beyond the scope of the review process to verify this information.

Response: We have double-checked the dates and details about the pandemic and confirmed that the details are correct.

7. Please make sure that your literature review is comprehensive and includes recent research on physical activity during the pandemic, such as this systematic review:

Wolf, S., Seiffer, B., Zeibig, J., Welkerling, J., Brokmeier, L., Atrott, B., Ehring, T., & Schuch, F. B. (2021). Is physical activity associated with less depression and anxiety during the COVID-19 pandemic? A rapid systematic review. Sports Medicine (Auckland), 51(8), 1771-1783. https://doi.org/10.1007/s40279-021-01468-z

Response: Thanks for the comment. We have conducted a more comprehensive literature review and added the reference suggested by the Editor and some more recent studies in China in the revised manuscript. The details of the changes are shown on pp. 4-5.
"A review of 21 studies about the effect of PA on mental health during the COVID-19 pandemic suggested that people who performed PA regularly with high volume and frequency and kept the PA routines stable would experience fewer symptoms of anxiety and depression [15]." (p.4)

"Although some studies have found that PA participation was positively associated with mental health among the Chinese adults during the COVID-19 pandemic [27,28], they did not specifically examine how the changes in PA and sitting time before and during the COVID-19 pandemic may affect mental health." (p.5)

Comments to the Author
Reviewer #1: PONE-D-21-27357

1.Abstract
The aim of the study is to examine physical and sedentary behaviour changes and online behavior on mental health and life satisfaction – but isn't this regarding a specific time frame of lockdown during COVID (based on your title – "during the covid-19 lockdown")? The abstract does not state that this study is specifically looking at participants behavioural and psychological outcomes during lockdown. Please clarify.

Powered by Editorial Manager® and ProduXion Manager® from Aries Systems Corporation

Response: Thanks for the comment. We have changed the title to "during the COVID-19 pandemic" since we share the same concern with the reviewer about the time frame of data collection. We have explained such a revision in response to the reviewer's comments #6 and #7. We stated explicitly that the study examined participants' behavioral and psychological outcomes during the COVID-19 pandemic in the revised Abstract.

"This study aimed to investigate the effects of physical and sedentary behavioral changes and online behavior during the COVID-19 pandemic on mental health and life satisfaction among the Chinese population."

2.Please clarify "first suffered from the COVID-19 pandemic between March 23 and April 9, 2020" - the introduction says COVID-19 was first reported in Dec 2019 and locked since January 23, 2020 and then relaxed March 23 and April 8 – How did they first suffer in March? Do you mean data was collected from March 23-April 9 regarding their experience with the lockdown from Jan-Mar 2020? Please clarify what March 23-April 9 is regarding as the article is about a lockdown period and this time frame is when lockdown was relaxed.

Response: Sorry for the confusion. We used "whose capital city Wuhan first suffered from the COVID-19 pandemic" to indicate that Hubei was the epicenter of the initial outbreak of the COVID-19 pandemic. To avoid confusion, we have deleted "whose capital city Wuhan first suffered from the COVID-19 pandemic" in the revised Abstract. The method section in the Abstract now reads:

"The data were obtained from a cross-sectional survey of 2145 residents aged between 18 and 80 in Hubei province, China between March 23, 2020, and April 9, 2020."

3.Introduction
Page 4 – you mention how the internet could enhance mental health (access to information, enabling people to work, study and shop online) – what about the ability for people to connect with loved ones/friends via online platforms (e.g., zoom, skype, facetime), especially during a lockdown. This would allow people to still "see" and talk to others during a lockdown. This could also help mental health. Also, gaming isn't necessarily a bad thing as people can game and chat with others which can be social, fun and something to do when in a lockdown. I'm not saying gaming could have negative effects, but it can also have good effects (specific to a lockdown).

Response: Thanks for the comment. We have included such a variable in our study. Our measure of online communication refers to the contact with family and friends via online communication tools (e.g., WeChat). WeChat is the most widely used communication tool in China, enabling people to see and talk to others. Also, we agree with the reviewer that online entertainment, such as gaming, could be a stress coping strategy and positively affect mental health during the pandemic. Our results actually support such an argument. The revised manuscript has included a hypothesis about Internet use and mental wellbeing. The corresponding changes are:

"(4) Internet use may be positively associated with mental wellbeing and life satisfaction during the COVID-19 pandemic." (p.6)

4.Page 5 – "a study of 4,898 adolescents…" – this study is pre-COVID, correct?

Response: That study was conducted during the COVID-19 pandemic. We have clarified it in the revised manuscript.

"A study of 4,898 adolescents in China showed that higher PA levels were significantly related to lower negative mood scores and higher positive mood scores during the COVID-19 pandemic" (p.5).

5.The introduction doesn't emphasize that you are looking at the lockdown period of COVID. The article mentions "during the COVID-19 pandemic". Are you looking at the lockdown only or lockdown and non-lockdown periods during COVID? During a lockdown period, behavioural and psychological outcomes would be influenced more

(can't leave their homes) vs non-lockdown periods since people can leave their homes. Please specific what time frame you are looking at and why this time frame (lockdown only or both lockdown and non-lockdown periods, etc.). Also, make sure you clearly state this time frame throughout the manuscript. Also, this seems to be looking at the first lockdown which is also important to state.

Response: Thanks for the comment. We would like to clarify the timeline of the lockdown of Hubei province. Chinese authorities imposed a lockdown on Wuhan, the capital city of Hubei province, on January 23, 2020, and enforced similar measures in 16 neighboring cities in Hubei province, affecting approximately 57 million people (Xiong, 2020). The authorities relaxed the Hubei lockdown on March 23, 2020, and officially lifted the Wuhan lockdown on April 8, 2020, after no new deaths transpired for the first time (He, 2020). The study was conducted between March 23, 2020, and April 9, 2020, when the lockdowns in Hubei were relaxed and eventually lifted. We asked the participants to report their physical activity participation and sitting time in the past two weeks and the past month, respectively, which may be during the lockdown or non-lockdown periods. Thus, we thought it would be better to use "COVID-19 pandemic" instead of "COVID-19 lockdown". We have used such a term throughout the manuscript.

6."the data collection was carried out between March 23 and April 9, 2020, while Hubei's lockdowns were gradually eased. Such data creates a unique opportunity for us to investigate Hubei residents' behavioral changes and psychological wellbeing during the lockdown period." – based on this sentence, my impression is you collected data during a non-lockdown period for participants to retrospectively report their behavioural and psychological outcomes during a lockdown (from Jan-Mar?), is this correct? You are specifically looking at lockdown effects. Why not collect data during the lockdown if its online anyways? Why wait until it eased when you are interested in lockdown effects? Please clarify.

Response: In the revised manuscript, we did not emphasize that we are looking at the lockdown period of the pandemic. We provided the reason in response to the reviewer's comment #5.
7.Page 6 - Please specify the specific "online behaviors" you plan to examine in this study.

Response: Thanks for the suggestion. We have specified the "online behaviors" in the revised manuscript (p.6). The corresponding changes are:

"Besides, we examined the impact of different types of Internet use, including information seeking, work/study, buying products, entertainment, reading/online learning, communication with family and friends, and investment/financial management."

8.Methods
How did the participants get recruited? Any incentives for them to complete the survey?

Response: We have provided more information about subject recruitment in the revised manuscript (p.7).

"The online survey was carried out on a professional survey platform in China. The platform sent a notification to respondents in their sample bank with a link to allow access to the questionnaire. Only Hubei residents (with IP address locations in Hubei) can answer the questionnaire. No incentives were provided to the participants."

9.Page 8 – for online behavior, what do you mean communication (talking with family members via zoom?). Please provide an example. Also, you looked at 7 internet uses which mostly seem like positive examples (based on your introduction) – you mention not having a hypothesis, but you only looked at internet uses that could enhance mental health; thus I would hypothesis internet use will have positive effects, correct? That said, why not include the negative internet uses you mentioned in the introduction such as gambling, social media use, shopping? Also, im assuming "buying products" is referring to "shop online that regularizes their routines". Please provide an example of

Powered by Editorial Manager® and ProduXion Manager® from Aries Systems Corporation

buying products.

Response: Thanks for the questions. To clarify, communication in this study was measured by asking the respondents whether they communicated with family and friends via online communication tools (e.g., WeChat). WeChat is the most widely used communication app in China, which acted as critical infrastructure in fulfilling people's practical, emotional, and medical needs during the COVID lockdown in China (Qian & Hanser, 2020). We have clarified this in the revised manuscript as below.

"communication with family and friends via online communication tools (e.g., WeChat)" (p.9).

As the reviewer suggested, we have included an example for buying products in the revised manuscript as "buying products (e.g., groceries)" (p. 9).

10.Page 8 – Did you explain what "moderate to vigorous physical activity" means to the participant in the survey (e.g., moderate to vigorous physical activity means any activity that your heart rate increases and you are sweating) or leave it to the participant to interpret it? For example, someone may interpret physical activity as anything active (e.g., doing daily chores) within a day while someone else may interpret specific working out activities (e.g., running for an hour). As you can imagine, a daily chore may not be as "active" as someone who runs for an hour. You mention in the discussion that future studies should look at type of physical activity, which I agree is very important, but curious how much information was given to the participants regarding this question.

Response: We asked the participants about the frequency of doing moderate-to-vigorous physical activity (MVPA), followed by a brief explanation and some examples of MVPA. In the survey, MVPA refers to exercises such as running, playing ball, swimming, dancing, brisk walking, etc. for ≥10 minutes. Typically, physical activity needs to continue for at least 10 minutes to be considered a session of exercise. We have clarified the measure of "moderate to vigorous physical activity" in the revised manuscript as below:

"Participants reported the frequency of doing moderate-to-vigorous physical activity (MVPA) in 2019 and in the past month, followed by a brief explanation and some examples of MVPA. MVPA refers to exercises such as running, playing ball, swimming, dancing, brisk walking, etc., for ≥10 minutes, which cause breathing and heart rate to increase." (p.8)

11.Page 8 – "information seeking" – is this specific COVID information seeking? Or other types of information seeking? How did you word this question?

Response: Thanks for the question. The information seeking was measured by general information seeking behavior, not specific COVID-19 information seeking. We have clarified this in the revised manuscript (p.9).

"Seven main and conceptually different Internet uses were accessed, including general information seeking;"

12.Results
You have most of the information from Table 1 and 2 in text (e.g., "half of the respondents were female (51.28%)."). Thus, Tables 1 and 2 are redundant. I would put all the information in text and remove Tables 1 and 2. Please include the sample size for each variable (e.g., half of the respondents were female (n = X; 51.28%)). Or keep Table 1 and 2 but remove the text because it is redundant. If you keep the tables – add the sample size for each variable and separate the Mean (SD) data into one column and the % data into another column.

Response: Thanks for the suggestions. To be consistent with the reporting style in most studies, we decided to keep Tables 1 and 2. As the reviewer suggested, we have removed the statistics and only kept a very brief description of PA, sitting time and online behavior in the revised manuscript.

Powered by Editorial Manager® and ProduXion Manager® from Aries Systems Corporation

13.Page 10 - The "M =" are missing for online class, work and study, buying products, investment and financial management.

Response: Thanks for pointing this out. However, as the reviewer suggested in Comment #12, we have deleted these statistics as they are redundant.

14.Regression results – since you have table 3 with all the test statistics, you don't need to have the statistics in text. You can remove the test statistic information and just explain the findings, as you have done (i.e., remove the numbers from this section that are in Table 3).

Response: Thanks for the suggestion. We have removed the statistics in the text.

15.Most participants completed the survey alone online, but some participants completed via phone survey. Any differences between the style of data collection (e.g., participants could ask for clarification during a phone survey from the research assistant vs participants who completed the survey alone on a computer). Were research assistants asked to not provide any additional information but simply read the questions to the participants over the phone? How many completed the survey via phone vs online?

Response: Thanks for the questions. We have clarified the two modes of data collection in the revised manuscript as below:

"There are minimal differences between the two modes of data collection. Our research staff will be asked not to provide any additional information but simply read the questions to the participants." (p.7)

We also included the number of participants who completed the survey online or via telephone in the revised manuscript.

"After deleting cases with at least one missing value, the final sample included 2145 respondents, of which 1944 (90.63%) completed the online survey, and 201 (9.37%) completed the phone survey." (p.7)

16.Discussion
Page 13 – I would not assume communication referring to use of social media, unless the question was framed this way. I would assume communication meaning communicating with others via phone or zoom/skype etc. When you clarify what "communication" means in the methods, this may make sense though.

Response: Thanks for the question. We have clarified this measurement in the revised manuscript.

"communication with family and friends via online communication tools (e.g., WeChat)"

17.I believe it is important to state that this study is looking at a lockdown period which is very different from normal day to day life (pre- and post-COVID). Something that was good during a lockdown (e.g., can't work so they play video games all day for social interaction and something to do), may not be good when the lockdown is lifted (e.g., playing video games but should be at work). Thus, these findings are only relevant when a city/town are under lockdown.

Response: Thanks again for the comment. As we responded to the reviewer earlier, we would not emphasize that we are looking at the lockdown effects. We use "during the COVID-19 pandemic" throughout the manuscript.

18.Page 13 - please clarify what shopping means? Shopping for non-essentials (e.g., equipment) vs essentials (e.g., food)? Or does online shopping have a negative effect even when shopping for essentials (i.e., any type of shopping)? Though, I thought online shopping for essentials could give the impression of regularize daily routines which would be good for mental health? Please clarify.

Response: Thanks for the comment. We did not specify types of shopping in the

	survey. It could be shopping for essentials or non-essentials.<br><br>19.Page 14 – another potential limitation – participants had to retrospectively report their physical activity and sitting time prior to COVID (especially 2019 MVPA rates) which could be inaccurate since it was based on memory (especially among older adults).<br><br>Response: Thanks for pointing this out. We have added the reporting bias in the limitation of the revised manuscript. The corresponding revisions are shown below:<br><br>"Moreover, we asked participants to retrospectively report their physical activity and sitting time prior to the COVID-19 pandemic, which may be subject to recall bias due to inaccurate memory. Future studies may use objective or direct measures of physical activity to increase precision and validate the self-report measures." (p.15)<br><br>References:<br>He J. China's State Machinery Will Beat Coronavirus Crisis, but at What Cost? South China Morning Post, February 1, 2020.<br><br>Xiong, Y. All major cities in China's Hubei province under lockdown. 2020. Retrieved from. https://edition.cnn.com/asia/live-news/coronavirus-outbreak-02-02-20-intl-hnk/h_9d7932db226e2e5109eabc8e4f5ae6c9<br><br>Qian Y, Hanser A. How did Wuhan residents cope with a 76-day lockdown?. Chinese Sociological Review. 2021 January 1;53(1):55-86.
**Additional Information:**	
**Question**	**Response**
**Financial Disclosure**<br><br>Enter a financial disclosure statement that describes the sources of funding for the work included in this submission. Review the submission guidelines for detailed requirements. View published research articles from *PLOS ONE* for specific examples.<br><br>This statement is required for submission and **will appear in the published article** if the submission is accepted. Please make sure it is accurate.	This work was supported by the National Social Science Fund of China (grant no. 16ZDA079).

Powered by Editorial Manager® and ProduXion Manager® from Aries Systems Corporation

**Unfunded studies**<br>Enter: *The author(s) received no specific funding for this work.*<br><br>**Funded studies**<br>Enter a statement with the following details:<br>• Initials of the authors who received each award<br>• Grant numbers awarded to each author<br>• The full name of each funder<br>• URL of each funder website<br>• Did the sponsors or funders play any role in the study design, data collection and analysis, decision to publish, or preparation of the manuscript?<br>• **NO** - Include this sentence at the end of your statement: *The funders had no role in study design, data collection and analysis, decision to publish, or preparation of the manuscript.*<br>• **YES** - Specify the role(s) played.<br><br>* typeset	
**Competing Interests**<br><br>Use the instructions below to enter a competing interest statement for this submission. On behalf of all authors, disclose any competing interests that could be perceived to bias this work—acknowledging all financial support and any other relevant financial or non-financial competing interests.<br><br>This statement is required for submission and **will appear in the published article** if the submission is accepted. Please make sure it is accurate and that any funding sources listed in your Funding Information later in the submission form are also declared in your Financial Disclosure statement.<br><br>View published research articles from *PLOS ONE* for specific examples.	The authors have declared that no competing interests exist.

**NO authors have competing interests**<br><br>Enter: *The authors have declared that no competing interests exist.*<br><br>**Authors with competing interests**<br><br>Enter competing interest details beginning with this statement:<br><br>*I have read the journal's policy and the authors of this manuscript have the following competing interests: [insert competing interests here]*<br><br>* typeset	
**Ethics Statement**<br><br>Enter an ethics statement for this submission. This statement is required if the study involved:<br><br>• Human participants<br>• Human specimens or tissue<br>• Vertebrate animals or cephalopods<br>• Vertebrate embryos or tissues<br>• Field research<br><br>Write "N/A" if the submission does not require an ethics statement.<br><br>General guidance is provided below. Consult the submission guidelines for detailed instructions. **Make sure that all information entered here is included in the Methods section of the manuscript.**	All procedures performed in studies involving human participants were in accordance with the ethical standards of the institutional and/or national research committee (Chinese Academy of Social Science) and with the 1964 Helsinki declaration and its later amendments or comparable ethical standards.

Powered by Editorial Manager® and ProduXion Manager® from Aries Systems Corporation

**Format for specific study types**<br><br>**Human Subject Research (involving human participants and/or tissue)**<br>• Give the name of the institutional review board or ethics committee that approved the study<br>• Include the approval number and/or a statement indicating approval of this research<br>• Indicate the form of consent obtained (written/oral) or the reason that consent was not obtained (e.g. the data were analyzed anonymously)<br><br>**Animal Research (involving vertebrate animals, embryos or tissues)**<br>• Provide the name of the Institutional Animal Care and Use Committee (IACUC) or other relevant ethics board that reviewed the study protocol, and indicate whether they approved this research or granted a formal waiver of ethical approval<br>• Include an approval number if one was obtained<br>• If the study involved *non-human primates*, add *additional details* about animal welfare and steps taken to ameliorate suffering<br>• If anesthesia, euthanasia, or any kind of animal sacrifice is part of the study, include briefly which substances and/or methods were applied<br><br>**Field Research**<br><br>Include the following details if this study involves the collection of plant, animal, or other materials from a natural setting:<br>• Field permit number<br>• Name of the institution or relevant body that granted permission	
**Data Availability**<br><br>Authors are required to make all data underlying the findings described fully available, without restriction, and from the time of publication. PLOS allows rare exceptions to address legal and ethical concerns. See the PLOS Data Policy and FAQ for detailed information.	No - some restrictions will apply

Powered by Editorial Manager® and ProduXion Manager® from Aries Systems Corporation

A Data Availability Statement describing where the data can be found is required at submission. Your answers to this question constitute the Data Availability Statement and **will be published in the article**, if accepted.<br><br>**Important:** Stating 'data available on request from the author' is not sufficient. If your data are only available upon request, select 'No' for the first question and explain your exceptional situation in the text box.<br><br>Do the authors confirm that all data underlying the findings described in their manuscript are fully available without restriction?	
**Describe where the data may be found in full sentences. If you are copying our sample text, replace any instances of XXX with the appropriate details.**<br><br>• If the data are **held or will be held in a public repository**, include URLs, accession numbers or DOIs. If this information will only be available after acceptance, indicate this by ticking the box below. For example: *All XXX files are available from the XXX database (accession number(s) XXX, XXX.*).<br>• If the data are all contained **within the manuscript and/or Supporting Information files**, enter the following: *All relevant data are within the manuscript and its Supporting Information files.*<br>• If neither of these applies but you are able to provide **details of access elsewhere**, with or without limitations, please do so. For example:<br><br>*Data cannot be shared publicly because of [XXX]. Data are available from the XXX Institutional Data Access / Ethics Committee (contact via XXX) for researchers who meet the criteria for access to confidential data.*<br><br>*The data underlying the results presented in the study are available from (include the name of the third party*	Data cannot be shared publicly because of data protection regulation. Data are available from the Chinese Social Quality Data Archive for researchers who meet the criteria for access to confidential data. The data are available for research upon reasonable request and with permission from the Chinese Social Quality Data Archive: http://csqr.cass.cn/index.jsp

and contact information or URL).<br>• This text is appropriate if the data are owned by a third party and authors do not have permission to share the data.<br><br>* typeset	
Additional data availability information:	

Powered by Editorial Manager® and ProduXion Manager® from Aries Systems Corporation

Dear Editor and reviewer,

Thank you very much for your valuable comments on our original manuscript. We have addressed all the comments raised by the reviewer and the Editor. Accordingly, we made extensive revisions to the manuscript based on the comments. We have submitted the revised manuscript highlighting changes in yellow and a clean version. We responded to each comment. Please feel free to contact us if you have further comments.

Thank you for your time!

Yours,
Dr. Xi Chen

Manuscript Click here to access/download;Manuscript;Manuscript.docx

Effects of changes in physical and sedentary behaviors on mental health and life satisfaction during the COVID-19 pandemic: Evidence from China

Xi Chen[1*]

Haiyan Gao[2]

Binbin Shu[3]

Yuchun Zou[2]

[1] The Jockey Club School of Public Health and Primary Care, The Chinese University of Hong Kong

[2] Faculty of Humanities and Social Sciences, Beijing University of Technology

[3] Department of Sociology, Shenzhen University, China

[4] Institute of Sociology, Chinese Academy of Social Sciences, China

***Author for correspondence:**

Xi Chen

Research Assistant Professor

The Jockey Club School of Public Health and Primary Care

The Chinese University of Hong Kong

Email: celiaxichen@cuhk.edu.hk (XC)

Address: Room 419, School of Public Health Building, Prince of Wales Hospital, Shatin, Hong Kong

// Abstract

Background: While restriction measures are critical in containing the COVID-19 outbreak, limited studies have investigated the behavioral and psychological impact of these measures. This study aimed to investigate the effects of physical and sedentary behavioral changes and online behavior during the COVID-19 pandemic on mental health and life satisfaction among the Chinese population.

Methods: The data were obtained from a cross-sectional survey of 2145 residents aged between 18 and 80 in Hubei province, China between March 23, 2020, and April 9, 2020.

Results: Participants who had high frequencies of physical activities before or during the COVID-19 outbreak exhibited higher levels of life satisfaction. Participants who increased their sitting time during the pandemic or kept sitting for more than eight hours before and during the pandemic reported worse mental health than those who maintained less sedentary behavior. Besides, participants who used the Internet for information seeking, communication, and entertainment more frequently reported better mental health and life satisfaction. In contrast, there was a positive association between commercial use of the Internet and symptoms of mental disorders

Conclusion: Given the link between physical and sedentary behavioral changes with worse mental wellbeing, strategies to reduce sedentariness and increase physical activity during the COVID-19 pandemic are needed.

Keywords: physical activity, sedentary behavior, mental health, life satisfaction, COVID-19 pandemic, China

Introduction

Mental health during the COVID-19 pandemic among the general population in China

The COVID-19 pandemic has heavily impacted many societies and economies. The total confirmed infection cases had reached over 483 million, and the death toll hit more than 6.1 million by March 30, 2022.[1] To control the spreading of COVID-19, governments worldwide have implemented various restriction measures ranging from social distancing to societal shutdown.[2] While these measures are critical in containing the outbreak, they may compromise individuals' mental health and health activities.[2] [3]

The outbreak of COVID-19 was first reported in Wuhan, the capital city of Hubei province, China, in December 2019. Authorities have put Wuhan and 16 neighboring cities in Hubei under lockdown since January 23, 2020. During the following weeks, COVID-19 cases increased rapidly and overwhelmed the health care system, especially in Wuhan. The situation improved when the Chinese government built massive temporary medical facilities to house COVID patients and transferred a large number of medical personnel to Hubei. The lockdowns were relaxed since March 23, 2020, and on April 8, 2020, the lockdown in Wuhan (the hardest-hit city in China) was officially lifted. Given the unprecedented social distancing measures, numerous studies have reported a high prevalence of mental health problems among Chinese adults during the COVID-19 pandemic.[4] [5] [6] However, most prior studies focused on sociodemographic backgrounds, risk perceptions, and attitudes toward COVID-19 as risk factors for psychological distress.[7] [8] Limited studies have investigated the effect of pandemic-related behavioral changes on population mental health.

Mental health consequences of physical activity, sedentary behavior, and online activities

Numerous studies have shown that the COVID-19-related restriction measures disrupted daily routines and impacted lifestyle activities, including participation in sports and physical activity (PA) [9] [10]. The decline in PA was accompanied by increased sedentary (sitting) behavior [11]. Given the consistent benefits of regular PA for mental health [12] [13] [14], reductions in PA are likely to exacerbate COVID-19-fueled psychological distress. A review of 21 studies about the effect of PA on mental health during the CCOVID-19 pandemic suggested that people who performed PA regularly with high volume and frequency and kept the PA routines stable would experience fewer symptoms of anxiety and depression [15].

Besides, accumulating evidence has revealed that, independent of PA levels, sedentary behavior is associated with mental health [16] [17]. Several longitudinal investigations have shown that self-reported sedentary time predicts future mental health and wellbeing [18] [19]. A longitudinal study of populations in France and Switzerland found that sedentary behavior increased during the lockdown period of COVID-19, which was associated with poorer physical health, mental health, and subjective vitality [20]. Increasing sitting time during the COVID-19 pandemic may be a risk factor for mental health and life satisfaction.

Moreover, COVID-19-related measures such as closing down schools, workplaces, and entertainment outlets coupled with self-quarantine have led to an unprecedented level of Internet and digital technology use. People move almost every aspect of their daily activities to cyberspace at an accelerating speed, as a “new normal” in the COVID-19 era. However, it remains unclear whether and how Internet usage would exacerbate or alleviate COVID-19-fueled psychological distress. On the one hand, the Internet could enhance mental health by providing access to the latest information and enabling people to work, study, and shop online that regularizes their routines during quarantines. On the other hand, excessive engagement in specific online activities such as gambling, gaming, social

media use, and shopping may result in severe problems and elevate the risk of disordered or addictive use of the Internet [21] [22]. Given the mixed evidence regarding the effects of Internet usage on mental health, more studies are warranted to investigate the impact of Internet usage on mental wellbeing during the COVID-19 pandemic.

To date, limited studies have examined the associations between changes in PA, sedentary behavior, and online behavior with mental health and quality of life among the Chinese population during the COVID-19 pandemic. A study of 4,898 adolescents in China showed that higher PA levels were significantly related to lower negative mood scores and higher positive mood scores during the COVID-19 pandemic [23]. More engagement in physical activities was also associated with reduced anxiety and depression among Chinese college students after adjusting for confounding demographic factors [24,25]. While providing important insights, these studies often focused on subpopulations (e.g., adolescents, college students), and thus their findings cannot be generalized to the general population. A study of the Chinese adult population found that compared with that before the lockdown, their time engaged in daily PA decreased and sedentary time increased, and PA is positively associated with life satisfaction [26]. However, the authors did not examine the effect of COVID-19-induced behavioral changes on adverse mental health outcomes. Although some studies have found that PA participation was positively associated with mental health among the Chinese adults during the COVID-19 pandemic [27,28], they did not specifically examine how the changes in PA and sitting time before and during the COVID-19 pandemic may affect mental health. Moreover, virtually no research has investigated how different types of online behavior during the pandemic may affect the Chinese population's psychological wellbeing during the COVID-19 pandemic.

The present study

Given the lack of research on the effects of COVID-19-induced behavioral changes on mental wellbeing among the Chinese population, this study aims to investigate the impacts of behavioral changes in PA, sedentary behavior, and online behavior on individual psychological wellbeing and life satisfaction

among residents in Hubei province, China, the original epicenter of the pandemic. The data collection was carried out between March 23, 2020, and April 9, 2020, while Hubei's lockdowns were gradually eased. Such data creates a unique opportunity for us to investigate Hubei residents' behavioral changes and psychological wellbeing during the pandemic. The findings of this study may improve our understanding and inform public health policies regarding the pattern and consequences of behavioral changes during the COVID-19 pandemic. Based on previous literature, we hypothesized that (1) the COVID-19 pandemic may be associated with changes in PA and sedentary behavior; (2) a decrease in PA may be negatively associated with mental health and life satisfaction; (3) an increase in sedentary behavior may have a negative effect on mental health and life satisfaction; and (4) Internet use may be positively associated with mental wellbeing and life satisfaction during the COVID-19 pandemic. In this study, we examined the impact of different types of Internet use, including information seeking, work/study, buying products, entertainment, reading/online learning, communication, and investment/financial management.

Methods

Data

The data came from the "Public Attitude toward The Novel Coronavirus Epidemic in Hubei Province" survey, which was carried out by the China Academy of Science and Technology Development Strategy, the Social Policy Research Institute at Renmin University, and the Institute of Sociology of the Chinese Academy of Social Sciences. The survey was conducted between March 23, 2020, and April 9, 2020. Authorities imposed a strict lockdown policy for cities in Hubei since January 23, 2020, and started to ease the lockdown since March 23, 2020, with the lockdown in Wuhan officially lifted on April 8, 2020.

The survey targeted all the residents aged between 18 and 80 in the urban and rural areas of Hubei. It was a combined online and phone survey. The online survey was conducted on a professional survey platform in China. The platform sent a notification to respondents in their sample bank with a link to

allow access to the questionnaire. Only Hubei residents (with IP address locations in Hubei) can answer the questionnaire. No incentives were provided to the participants. A supplemented phone survey was carried out by trained research assistants to reach populations with limited access to the Internet (e.g., aged over 65, rural population). There are minimal differences between the two modes of data collection. Our research staff will be asked not to provide any additional information but simply read the questions to the participants. Participants who did the online survey provided written consent. Participants who answered the questions via phone gave oral consent, and the interviewer signed a form pledging that he/she had gone through the proper procedures to obtain the verbal informed consent of the participant. The study was approved by the ethical committee of the Chinese Academy of Social Science, and all procedures were in accordance with the 1964 Helsinki declaration and its later amendments or comparable ethical standards. In total, 2355 participants completed the online or phone surveys. After deleting cases with at least one missing value, the final sample included 2145 respondents, of which 1944 (90.63%) completed the online survey, and 201 (9.37%) completed the phone survey.

Measurements

Symptoms of mental disorders were measured by the 12-item Chinese Health Questionnaire scale (CHQ-12), which was widely used for screening non-psychotic mental health problems in the general population. Developed by Cheng and Williams (1986), CHQ was adapted from the General Health Questionnaire [29] with the inclusion of culturally relevant items in Chinese societies. CHQ-12 has been validated in the general population in mainland China [30] [31], Taiwan [32] [33], and Chinese people in foreign countries [34] [35]. Ten items of CHQ-12 were negatively worded (e.g., “Been suffering from headache or pressure in your head”) and two items were positively worded (e.g., “Have you been getting along well with your family or friends recently?”). The responses for all items ranged from 0 = “not at all” to 3 = “very often.” The responses for positive ones were reversely coded so that higher values indicated higher levels of mental disorder. The internal validity of the scale was good (Chronbach’s alpha = 0.78). In this study, the cut-off point against the diagnosis of non-psychotic disorders was 13/14.

About 32.07% of the participants scored greater than or equal to 14 and could be regarded as having symptoms consistent with potential mental disorders.

Life satisfaction was assessed by the question, "In general, how satisfied are you with your life?" with a 5-point scale from 1 = "very dissatisfied" to 5 = "very satisfied." Many studies and large-scale surveys have used such a single-item life satisfaction measure [36] [37]. Prior research has confirmed the reliability of this single-item measure of life satisfaction by showing that it performed very similarly compared to the multiple-item Satisfaction with Life Scale (SWLS) and correlated with external variables (e.g., health and affect) to a similar degree [38] [39].

Physical activity. Participants reported the frequency of doing moderate-to-vigorous physical activity (MVPA) in 2019 and in the past month, followed by a brief explanation and some examples of MVPA. MVPA refers to exercises such as running, playing ball, swimming, dancing, brisk walking, etc., for ≥10 minutes, which cause breathing and heart rate to increase. The response categories ranged from "never" to "every day." Exercise training guidelines for a quarantine situation due to COVID-19 suggested that healthy or asymptomatic persons should do aerobic exercises for at least three sessions per week (four or five seem optimal) [41]. Participants were categorized as "maintaining high PA" if they did PA > two times a week, as "decreased PA" if they did PA for > two times a week before the COVID-19 pandemic but did not do so afterward, as "increased PA" if they increased PA to > two times a week during the pandemic, and as "maintaining low PA" if they did PA for less than two times a week before and during the pandemic.

Sitting time. Participants reported average daily time spent sitting before (i.e., in early January 2020) and during the COVID-19 pandemic. Previous studies suggested that the association of daily sitting time and most long-term health outcomes is not linear, with a weekday sitting time below 8 hours/day associated with better perceived mental health and quality of life [40]. Participants were then classified as "maintaining low sitting time" if they reported ≤8h/day of sitting at both time points, as "increasing sitting time" if they reported ≤ 8h/day of sitting before the pandemic but reported > 8h/day of sitting

after the pandemic, as “decreasing sitting time” if they reported > 8h/day of sitting before the pandemic but reported ≤ 8h/day of sitting after the pandemic, and as “maintaining high sitting time” if they reported > 8h/day of sitting at both time points.

Online behavior. Seven main and conceptually different types of Internet uses were accessed, including general information seeking; work; buying products (e.g., groceries); entertainment (e.g., playing online games, watching videos, listening to music); reading books/online class; communication with family and friends via online communication tools (e.g., WeChat); and investment and financial management. The responses ranged from 0 = “never” to 4 = “everyday”. Higher values indicated a higher frequency of using the Internet for the corresponding activity.

In addition, a series of sociodemographic variables were measured, including age (continuous), sex (male vs. female), education (middle school or below, high school, college or above), occupation (managerial/professional position, manual/service/part-time worker, or other), China Communist Party membership (Party member vs. non-Party member), monthly personal income (from no income to >8000 *yuan*), area (rural vs. urban), city (Wuhan vs. non-Wuhan).

Analysis

All the analyses were performed using Stata 16.0. Descriptive statistics were used to characterize the study population. Considering the variable of potential mental disorder was binary, we fit multiple logistic regression models to study the associations between changes in sitting time and PA during the COVID-19 pandemic and online behavior with symptoms of potential mental disorders. For the outcome variable of life satisfaction, we used OLS regression models to examine the effect of sedentary activity, PA, and online behavior after adjusting for sociodemographic variables. Variance inflation factors (VIFs) of the independent variables were estimated to check whether multicollinearity exists in the models. All the VIFs were below 2, suggesting that multicollinearity was not a significant concern.

Results

Table 1 displays the background characteristics of the respondents.

<Table 1 about here>

Table 2 shows the descriptive statistics of independent and dependent variables. Compared to the pre-COVID-19 period, about 4.66% of participants increased their sitting time, and approximately 15.10% reduced their PA during the pandemic. As for various online behavior, participants were most frequently to use the Internet for information seeking, followed by communication, entertainment, reading, and attending the online class, work and study. Relatively fewer participants used the Internet for commercial activities, including buying products and investment and financial management.

<Table 2 about here>

Table 3 presents the associations between physical activities and sedentary behavior changes, and various online behavior with depression (Models 1 and 2) and life satisfaction (Models 3 and 4) after adjusting for sociodemographic variables. As shown in Model 1, compared to participants who sit less than 8 hours before and after the pandemic, those who increased their sitting time during the pandemic and kept sitting for more than 8 hours a day at both time points were more likely to experience symptoms of mental disorders. However, changes in the frequency of PA were not associated with symptoms of mental disorders. Model 2 examined the impact of digital engagement and revealed that information seeking, entertainment, and communication activities were associated with lower levels of symptoms of mental disorders. Conversely, online commercial activities, including buying products and doing investment online were positively associated with symptoms of mental disorders. Compared to those who participated in lower levels of physical activity, participants who increased, decreased, or maintained high frequencies of physical activity exhibited higher levels of life satisfaction. In contrast to the mixed effect of digital engagement on depression, most online behaviors positively effect on life

satisfaction. Specifically, using the Internet for information seeking, study/work, reading books/online class, and communication were positively associated with life satisfaction. Other online behaviors, such as buying products, entertainment, and investment/financial management, were not significantly associated with life satisfaction.

<Table 3 about here>

Discussion

This study presents a timely investigation of the effects of behavioral changes (i.e., PA, sedentary behavior, and online behavior) induced by COVID-19-related restrictions on psychological wellbeing and life satisfaction among the general population in Hubei province, China, the original epicenter of the pandemic. With data obtained from 2145 Hubei residents during the pandemic, the findings showed (1) an expected decline in PA and an increase in sitting time during the pandemic; (2) an association between increased sitting time and symptoms of mental disorder; (3) higher life satisfaction for those doing more exercises; and (4) a mixed pattern of Internet uses and mental health and life satisfaction.

While it is well-documented that increased PA could reduce mental health problems [42] [43] [44], there is insufficient evidence regarding the effect of PA on positive mental health among the Chinese population. The findings of our study revealed that more involvement in PA during the COVID-19 pandemic might effectively improve life satisfaction among Hubei residents. PA could be associated with life satisfaction through fitness- and health-related adaptations that enhance physical and mental health. PA may also have a revitalizing effect that strengthens self-control and supports goal pursuits [45] [46]. Considering we live in times of great psychological and emotional fragility, it is even more essential to promote PA that may help people adapt to the crisis and gain more life satisfaction. Our findings suggest that those who have low frequencies of PA during COVID-19 social distancing may need to be targeted with support for their quality of life. Policymakers and stakeholders should develop health education and communication that emphasize PA to improve life satisfaction during an

infectious disease outbreak. As people have limited opportunities for doing PA outside their home during social distancing, future PA intervention to foster an Active and Healthy Confinement Lifestyle (AHCL) during a pandemic may adopt ICT solutions, such as home-based exercise games and fitness apps.

Besides, our findings indicated that the overall time spent in sedentary behavior is positively associated with symptoms of mental disorders. Sedentary behavior could be associated with mental disorders via several possible pathways. For example, screen-based sedentary behaviors (e.g., TV viewing) are likely to induce addiction [47], internalizing symptoms [48], and low sleeping quality [49] , potentially leading to heightened levels of mental distress. Further, engaging in sedentary behaviors may displace time spent in other activities such as household or work-related responsibilities or PA, which may be effective coping strategies for symptoms of mental disorders [50]. It is worth noting that specific sedentary behaviors may contribute to mental health in different ways. For example, a study found that specific screen-based recreation was associated with higher self-esteem among Australian adolescents in low-income communities [51]. Thus, future studies may investigate associations between different types of sedentary behavior and mental health.

Our findings showed that various Internet use types had different effects on mental health and life satisfaction during the COVID-19 pandemic among Hubei residents. To start with, online information-seeking behavior was associated with a lower likelihood of mental disorders and increased life satisfaction. Such findings seemed inconsistent with other studies showing that exposure to COVID-19-related information may increase psychological distress [52] [53]. The discrepancy between our study and others may be that we measured the general information-seeking behavior rather than COVID-19 specific information seeking. Future studies may further investigate whether information-seeking behavior in general and about COVID-19 has differential effects on psychological wellbeing.

Also, online communication activity played an important role in improving mental health and life satisfaction, which appeared contradictory to prior studies showing that online communication

(especially the use of social media) may deteriorate mental health due to its negative impact on face-to-face communications [54] [55]. Considering offline interactions were restricted by physical distancing and stay-at-home orders during the COVID-19 pandemic, online contact with friends and family may increase emotional support, decrease loneliness and social isolation, which leads to lower depression and improved life satisfaction. Besides, our results have shown that online entertainment can reduce symptoms consistent with potential mental disorders. Using the Internet to relax may be a positive coping strategy to alleviate feelings of stress and isolation [56]. Similarly, working and learning remotely during the pandemic may keep people connected with their co-workers and classmates, despite physical isolation, and thus were positively associated with life satisfaction. Using the Internet for work and study may also help regularize daily routines, which is essential for buffering the adverse impact of stress exposure during a crisis [57].

While respondents who used the Internet for information seeking, communication, study/work, reading books, and entertainment reported better mental health and life satisfaction, the commercial use of the Internet (e.g., shopping, investment) seemed to increase mental distress. We conjecture that the economic downturn and uncertainty caused by the pandemic may be why people who were involved in more online economic activities suffered more distress. Previous studies also found that online shopping may be a negative coping strategy for stress and was associated with increased symptoms of mental disorders [58] [59]. While online purchases were the dominant way for people to get groceries due to social distancing and stay-at-home orders, scholars have suggested setting a specific time and financial limits for online shopping to reduce the risk of disordered or addictive use [60].

Despite the significant findings, this study is not without limitations. First, due to the cross-sectional design employed in the present study, it cannot ascertain whether mental health problems lead to lower PA levels or whether lower levels of PA lead to mental health problems during the COVID-19 pandemic. Additional longitudinal studies are needed to infer the direction of the association. Second, our samples are not representative since it was mainly conducted online. However, considering in-person surveys would not be appropriate or possible during the COVID-19 pandemic, online surveys are arguably the

most feasible way to collect data in a timely matter. We have also supplemented the online survey with a telephone survey to reach groups with limited access to the Internet. Future studies may try various sampling methods to gain a population-based representative sample. Third, participants were asked to self-report their PA level, thus introducing self-reporting bias into the findings. Moreover, we asked participants to retrospectively report their physical activity and sitting time prior to the COVID-19 pandemic, which may be subject to recall bias due to inaccurate memory. Future studies may use objective or direct measures of physical activity to increase precision and validate the self-report measures. Lastly, due to the online survey's length limitation, we only measured the PA frequency and the total amount of sedentary behavior during the pandemic. Future studies may further specify the level and type of PA and sedentary behavior and their relationship with mental health and life satisfaction.

Conclusion

In conclusion, social distancing and quarantine measures to mitigate the COVID-19 pandemic may affect PA, mental health, and life satisfaction among the general population, with those experiencing a decrease in PA having lower life satisfaction, and those sitting more time reporting a higher likelihood of mental disorders. Besides, the increasing use of the Internet and digital technology throughout the pandemic also impacts individuals' mental health and life satisfaction. Our findings showed that participants who used the Internet for information seeking, communication, study/work, reading books, and entertainment more frequently reported better mental health and life satisfaction. In contrast, the commercial use of the Internet (e.g., shopping, investment) seemed to increase mental distress.

Acknowledgment

This work was supported by the National Social Science Fund of China (grant no. 16ZDA079). The funders had no role in study design, data collection and analysis, decision to publish, or preparation of the manuscript.

References:

[1] World Health Organization. WHO Coronavirus (COVID-19) Dashboard. Available at: https://covid19.who.int/ (accessed on March 30, 2022)

[2] Benke C, Autenrieth LK, Asselmann E, Pané-Farré CA. Lockdown, quarantine measures, and social distancing: Associations with depression, anxiety and distress at the beginning of the COVID-19 pandemic among adults from Germany. Psychiatry Research. 2020 Nov 1;293:113462.

[3] Brooks SK, Webster RK, Smith LE, Woodland L, Wessely S, Greenberg N, Rubin GJ. The psychological impact of quarantine and how to reduce it: rapid review of the evidence. The Lancet. 2020 Feb 26.

[4] Qiu J, Shen B, Zhao M, Wang Z, Xie B, Xu Y. A nationwide survey of psychological distress among Chinese people in the COVID-19 epidemic: implications and policy recommendations. General psychiatry. 2020;33(2).

[5] Tang W, Hu T, Hu B, Jin C, Wang G, Xie C, Chen S, Xu J. Prevalence and correlates of PTSD and depressive symptoms one month after the outbreak of the COVID-19 epidemic in a sample of home-quarantined Chinese university students. Journal of affective disorders. 2020 May 13.

[6] Xin M, Luo S, She R, Yu Y, Li L, Wang S, Ma L, Tao F, Zhang J, Zhao J, Li L. Negative cognitive and psychological correlates of mandatory quarantine during the initial COVID-19 outbreak in China. American Psychologist. 2020 Jul;75(5):607.

[7] Kwok KO, Li KK, Chan HH, Yi YY, Tang A, Wei WI, Wong YS. Community responses during the early phase of the COVID-19 epidemic in Hong Kong: risk perception, information exposure and preventive measures. MedRxiv. 2020 Jan 1.

[8] Xiong J, Lipsitz O, Nasri F, Lui LM, Gill H, Phan L, Chen-Li D, Iacobucci M, Ho R, Majeed A, McIntyre RS. Impact of COVID-19 pandemic on mental health in the general population: A systematic review. Journal of affective disorders. 2020 Aug 8.

[9] Ammar A, Brach M, Trabelsi K, Chtourou H, Boukhris O, Masmoudi L, Bouaziz B, Bentlage E, How D, Ahmed M. Effects of COVID-19 home confinement on social participation and life satisfaction: Preliminary results of the ECLB-COVID19 international online-survey. medRxiv. 2020.

[10] Barwais FA, Cuddihy TF, Tomson LM. Physical activity, sedentary behavior and total wellness changes among sedentary adults: a 4-week randomized controlled trial. Health and quality of life outcomes. 2013 Dec 1;11(1):183.

[11] Hall G, Laddu DR, Phillips SA, Lavie CJ, Arena R. A tale of two pandemics: How will COVID-19 and global trends in physical inactivity and sedentary behavior affect one another?. Progress in Cardiovascular Diseases. 2020 Apr 8.

[12] Taylor CB, Sallis JF, Needle R. The relation of physical activity and exercise to mental health. Public health reports. 1985 Mar;100(2):195.

[13] Deslandes A, Moraes H, Ferreira C, Veiga H, Silveira H, Mouta R, Pompeu FA, Coutinho ES, Laks J. Exercise and mental health: many reasons to move. Neuropsychobiology. 2009;59(4):191-8.

[14] Ashdown-Franks G, Firth J, Carney R, Carvalho AF, Hallgren M, Koyanagi A, Rosenbaum S, Schuch FB, Smith L, Solmi M, Vancampfort D. Exercise as medicine for mental and substance use disorders: a meta-review of the benefits for neuropsychiatric and cognitive outcomes. Sports Medicine. 2020 Jan 1:1-20.

[15] Wolf S, Seiffer B, Zeibig JM, Welkerling J, Brokmeier L, Atrott B, Ehring T, Schuch FB. Is physical activity associated with less depression and anxiety during the COVID-19 pandemic? A rapid systematic review. Sports Medicine. 2021 Aug;51(8):1771-83.

[16] Salmon P. Effects of physical exercise on anxiety, depression, and sensitivity to stress: a unifying theory. Clinical psychology review. 2001 Feb 1;21(1):33-61.

[17] Wilcox S, Dowda M, Leviton LC, Bartlett-Prescott J, Bazzarre T, Campbell-Voytal K, Carpenter RA, Castro CM, Dowdy D, Dunn AL, Griffin SF. Active for life: final results from the translation of two physical activity programs. American journal of preventive medicine. 2008 Oct 1;35(4):340-51.

[18] Lucas M, Mekary R, Pan A, Mirzaei F, O'Reilly ÉJ, Willett WC, Koenen K, Okereke OI, Ascherio A. Relation between clinical depression risk and physical activity and time spent watching television in older women: a 10-year prospective follow-up study. American journal of epidemiology. 2011 Nov 1;174(9):1017-27.

[19] Sanchez-Villegas A, Ara I, Guillen-Grima F, Bes-Rastrollo M, Varo-Cenarruzabeitia JJ, Martinez-Gonzalez MA. Physical activity, sedentary index, and mental disorders in the SUN cohort study. Medicine & Science in Sports & Exercise. 2008 May 1;40(5):827-34.

[20] Cheval B, Sivaramakrishnan H, Maltagliati S, Fessler L, Forestier C, Sarrazin P, Orsholits D, Chalabaev A, Sander D, Ntoumanis N, Boisgontier MP. Relationships between changes in self-reported physical activity, sedentary behaviour and health during the coronavirus (COVID-19) pandemic in France and Switzerland. Journal of sports sciences. 2020 Oct 31:1-6.

[21] Fineberg NA, Apergis-Schoute AM, Vaghi MM, Banca P, Gillan CM, Voon V, Chamberlain SR, Cinosi E, Reid J, Shahper S, Bullmore ET. Mapping compulsivity in the DSM-5 obsessive compulsive and related disorders: cognitive domains, neural circuitry, and treatment. International Journal of Neuropsychopharmacology. 2018 Jan;21(1):42-58.

[22] Peeters M, Koning I, Lemmens J, Eijnden RV. Normative, passionate, or problematic? Identification of adolescent gamer subtypes over time. Journal of behavioral addictions. 2019 Sep;8(3):574-85.

[23] Kang S, Sun Y, Zhang X, Sun F, Wang B, Zhu W. Is Physical Activity Associated with Mental Health among Chinese Adolescents during Isolation in COVID-19 Pandemic?. Journal of Epidemiology and Global Health. 2020 Sep.

[24] Xiang MQ, Tan XM, Sun J, Yang HY, Zhao XP, Liu L, Hou XH, Hu M. Relationship of physical activity with anxiety and depression symptoms in Chinese college students during the COVID-19 outbreak. Frontiers in Psychology. 2020;11.

[25] Zhang Y, Wu X, Tao S, Li S, Ma L, Yu Y, Sun G, Li T, Tao F. Associations between screen time, physical activity, and depressive symptoms during the 2019 coronavirus disease (COVID-19) outbreak among Chinese college students. Environmental Health and Preventive Medicine. 2021 Dec;26(1):1-2.

[26] Wang X, Lei SM, Le S, Yang Y, Zhang B, Yao W, Gao Z, Cheng S. Bidirectional influence of the COVID-19 pandemic lockdowns on health behaviors and quality of life among Chinese adults. International Journal of Environmental Research and Public Health. 2020 Jan;17(15):5575.

[27] Qi M, Li P, Moyle W, Weeks B, Jones C. Physical activity, health-related quality of life, and stress among the Chinese adult population during the COVID-19 pandemic. International journal of environmental research and public health. 2020 Jan;17(18):6494.

[28] Nie Y, Ma Y, Wu Y, Li J, Liu T, Zhang C, Lv C, Zhu J. Association between physical exercise and mental health during the COVID-19 outbreak in China: a nationwide cross-sectional study. Frontiers in Psychiatry. 2021:1381.

[29] Goldberg DP. User's guide to the General Health Questionnaire. Windsor. 1988.

[30] Yang TZ, Huang L, Wu ZY. The application of Chinese health questionnaire for mental disorder screening in community settings in mainland China. Zhonghua Liu Xing Bing Xue Za Zhi= Zhonghua Liuxingbingxue Zazhi. 2003 Sep 1;24(9):769-73.

[31] Zhang CC, Hou LH, Zheng X, Lu J, Zou JY, Qian Y, Yang TZ. Factor structure of 12 items in the Chinese Health Questionnaire among the elderly population in mainland China. Public Health. 2020 Oct 1;187:143-9.

[32] Cheng TA, Williams P. The design and development of a screening questionnaire (CHQ) for use in community studies of mental disorders in Taiwan. Psychological medicine. 1986 May;16(2):415-22.

[33] Cheng TA, Wu JT, Chong MY, Williams P. Internal consistency and factor structure of the Chinese Health Questionnaire. Acta Psychiatrica Scandinavica. 1990 Oct;82(4):304-8.

[34] Pan PC, Goldberg DP. A comparison of the validity of GHQ-12 and CHQ-12 in Chinese primary care patients in Manchester. Psychological Medicine. 1990 Nov;20(4):931-40.

[35] Barbato A, Aresu A, Battino RN, Troisi E, Tettamanti M, Parabiaghi A. A tool for cross-cultural psychiatry research: a pilot study of adaptation of 12-item Chinese Health Questionnaire (CHQ-12) for Chinese population in Italy. Rivista di psichiatria. 2009;44(4):249-57.

[36] Wagner GG, Joachim RF, Jürgen S. The German Socio-Economic Panel Study (SOEP)-Scope, Evolution and Enhancements. Schmollers Jahrbuch: Journal of Applied Social Science Studies. 2007;127(1):139–169.

[37] Taylor MF, Brice J, Buck N, Prentice-Lane E. British Household Panel Survey user manual volume A: Introduction, technical report, and appendices. Colchester: University of Essex; 2009. Available at: http://iserwww.essex.ac.uk/ulsc/bhps/doc/

[38] Kobau R, Sniezek J, Zack MM, Lucas RE, Burns A. Well-Being Assessment: An Evaluation of Well-Being Scales for Public Health and Population Estimates of Well-Being among US Adults. Applied Psychology: Health and Well-Being. 2010;2(3):272–297.

[39] Cheung F, Lucas RE. Assessing the validity of single-item life satisfaction measures: Results from three large samples. Quality of Life research. 2014 Dec 1;23(10):2809-18.

[40] Gibson AM, Muggeridge DJ, Hughes AR, Kelly L, Kirk A. An examination of objectively-measured sedentary behavior and mental wellbeing in adults across week days and weekends. PloS one. 2017 Sep 21;12(9):e0185143.

[41] Khoramipour K, Basereh A, Hekmatikar AA, Castell L, Ruhee RT, Suzuki K. Physical activity and nutrition guidelines to help with the fight against COVID-19. Journal of Sports Sciences. 2020 Aug 27:1-7.

[42] McDowell CP, Dishman RK, Gordon BR, Herring MP. Physical activity and anxiety: a systematic review and meta-analysis of prospective cohort studies. American journal of preventive medicine. 2019 Oct 1;57(4):545-56.

[43] Schuch FB, Vancampfort D, Firth J, Rosenbaum S, Ward PB, Silva ES, Hallgren M, Ponce De Leon A, Dunn AL, Deslandes AC, Fleck MP. Physical activity and incident depression: a meta-analysis of prospective cohort studies. American Journal of Psychiatry. 2018 Jul 1;175(7):631-48.

[44] White RL, Babic MJ, Parker PD, Lubans DR, Astell-Burt T, Lonsdale C. Domain-specific physical activity and mental health: a meta-analysis. American journal of preventive medicine. 2017 May 1;52(5):653-66.

[45] Penedo FJ, Dahn JR. Exercise and wellbeing: a review of mental and physical health benefits associated with physical activity. Current opinion in psychiatry. 2005 Mar 1;18(2):189-93.

[46] Rejeski WJ, Mihalko SL. Physical activity and quality of life in older adults. The Journals of Gerontology Series A: Biological sciences and medical sciences. 2001 Oct 1;56(suppl_2):23-35.

[47] Cheng YS, Tseng PT, Lin PY, Chen TY, Stubbs B, Carvalho AF, Wu CK, Chen YW, Wu MK. Internet addiction and its relationship with suicidal behaviors: a meta-analysis of multinational observational studies. The Journal of clinical psychiatry. 2018 Jun 5;79(4):0-.

[48] Zink J, Belcher BR, Imm K, Leventhal AM. The relationship between screen-based sedentary behaviors and symptoms of depression and anxiety in youth: a systematic review of moderating variables. BMC public health. 2020 Dec;20:1-37.

[49]Fossum IN, Nordnes LT, Storemark SS, Bjorvatn B, Pallesen S. The association between use of electronic media in bed before going to sleep and insomnia symptoms, daytime sleepiness, morningness, and chronotype. Behavioral sleep medicine. 2014 Sep 3;12(5):343-57.

[50] Teychenne M, Hinkley T. Associations between screen-based sedentary behaviour and anxiety symptoms in mothers with young children. PLoS One. 2016 May 18;11(5):e0155696.

[51]Nihill GF, Lubans DR, Plotnikoff RC. Associations between sedentary behavior and self-esteem in adolescent girls from schools in low-income communities. Mental Health and Physical Activity. 2013;6(1):30-5.

[52] Chao M, Chen X, Liu T, Yang H, Hall BJ. Psychological distress and state boredom during the COVID-19 outbreak in China: the role of meaning in life and media use. European journal of psychotraumatology. 2020 Dec 31;11(1):1769379.

[53] Gao J, Zheng P, Jia Y, Chen H, Mao Y, Chen S, Wang Y, Fu H, Dai J. Mental health problems and social media exposure during COVID-19 outbreak. Plos one. 2020;15(4):e0231924.

[54] Caplan SE. Preference for online social interaction: A theory of problematic Internet use and psychosocial wellbeing. Communication research. 2003 Dec;30(6):625-48.

[55] Kim J, LaRose R, Peng W. Loneliness as the cause and the effect of problematic Internet use: The relationship between Internet use and psychological wellbeing. Cyberpsychology & behavior. 2009 Aug 1;12(4):451-5.

[56] Pressman SD, Matthews KA, Cohen S, Martire LM, Scheier M, Baum A, Schulz R. Association of enjoyable leisure activities with psychological and physical wellbeing. Psychosomatic medicine. 2009 Sep;71(7):725.

[57] Hou WK, Lai FT, Ben-Ezra M, Goodwin R. Regularizing daily routines for mental health during and after the COVID-19 pandemic. Journal of Global Health. 2020 Dec;10(2).

[58] Morgan C, Cotten SR. The relationship between Internet activities and depressive symptoms in a sample of college freshmen. CyberPsychology & Behavior. 2003 Apr 1;6(2):133-42.

[59]Norberg MM, Crone C, Kwok C, Grisham JR. Anxious attachment and excessive acquisition: The mediating roles of anthropomorphism and distress intolerance. Journal of Behavioral Addictions. 2018 Mar;7(1):171-80..

[60] Király O, Potenza MN, Stein DJ, King DL, Hodgins DC, Saunders JB, Griffiths MD, Gjoneska B, Billieux J, Brand M, Abbott MW. Preventing problematic internet use during the COVID-19 pandemic: Consensus guidance. Comprehensive Psychiatry. 2020 May 12:152180.

Table 1. Descriptive statistics of background variables

Variable	N (%)	
Age, Mean (SD)	33.70 (12.80)	
Sex		
Male	1045	(48.72)
Female	1100	(51.28)
Education		
Middle school or below	257	(11.98)
High school	483	(22.52)
College or above	1405	(65.50)
Occupation		
Managerial/professional position	495	(23.08)
Manual/service worker	1282	(59.77)
Other	368	(17.16)
Communist Party membership		
Party member	343	(15.99)
Non-Party member	1802	(84.01)
Monthly income		
No income	390	(18.18)
≤2000	451	(21.03)
2001-4000	642	(29.93)
4001-6000	394	(18.37)
6001-8000	159	(7.41)
>8001	108	(5.08)
Area		
Rural	165	(7.69)
Urban	1980	(92.31)
City		
Wuhan	541	(25.22)
Non-Wuhan	1604	(74.78)

Table 2. Descriptive statistics of dependent and independent variables

Variable	N (%)	Mean (SD)
Mental Health (CHQ-12)		
Probable mental disorders	688 (32.07)	
No probable mental disorders	1457 (67.93)	
Life satisfaction		3.89 (0.94)
Physical activity		
Maintained low	229 (10.68)	
Increased	117 (5.45)	
Decreased	324 (15.10)	
Maintained high	1475 (68.76)	
Sitting time		
Maintained low	1922 (89.60)	
Increased	100 (4.66)	
Decreased	53 (2.47)	
Maintained high	70 (3.26)	
Online behavior		
Information seeking		3.55 (0.80)
Work/study		2.91 (1.27)
Buying products		2.60 (1.06)
Entertainment		3.33 (0.91)
Reading/online learning		3.04 (1.17)
Communication		3.36 (0.94)
Investment/financial management		1.95 (1.35)

Table 3. Adjusted associations between self-reported changes in behavior before and during the COVID-19 pandemic and mental health and life satisfaction.

	Probable mental disorders				Life satisfaction			
	Model 1		Model 2		Model 3		Model 4	
	OR	95% CI	OR	95% CI	b	95% CI	b	95% CI
Physical activity								
Maintained low	Reference				Reference			
Increased	1.492	[0.924,2.409]			0.246*	[0.040,0.453]		
Decreased	0.721	[0.488,1.065]			0.238**	[0.081,0.395]		
Maintained high	1.322	[0.966,1.808]			0.425***	[0.294,0.555]		
Sitting time								
Maintained low	Reference				Reference			
Increased	2.384***	[1.579,3.600]			-0.010	[-0.197,0.176]		
Decreased	1.031	[0.564,1.884]			-0.015	[-0.268,0.237]		
Maintained high	2.022**	[1.229,3.324]			-0.188	[-0.411,0.035]		
Online behavior								
Information seeking			0.666***	[0.591,0.752]			0.095***	[0.043,0.148]
Work/study			0.940	[0.863,1.024]			0.052**	[0.018,0.086]
Buying products			1.217***	[1.093,1.354]			0.039	[-0.004,0.081]
Entertainment			0.878*	[0.784,0.984]			0.012	[-0.036,0.060]
Reading/online learning			0.953	[0.871,1.042]			0.054**	[0.018,0.091]
Communication			0.857**	[0.766,0.958]			0.048*	[0.001,0.096]
Investment/financial management			1.181***	[1.091,1.277]			-0.006	[-0.038,0.027]
Sex								
Male	Reference		Reference		Reference		Reference	
Female	0.958	[0.795,1.153]	0.981	[0.811,1.186]	-0.036	[-0.115,0.043]	-0.039	[-0.117,0.040]
Age	1.004	[0.996,1.011]	1.003	[0.995,1.011]	-0.001	[-0.004,0.003]	0.000	[-0.003,0.003]
Education								
Middle school or below	Reference		Reference		Reference		Reference	
High school	1.028	[0.737,1.434]	1.042	[0.741,1.464]	0.078	[-0.063,0.220]	0.077	[-0.063,0.218]
College or above	0.980	[0.728,1.319]	0.961	[0.709,1.302]	0.041	[-0.085,0.167]	0.031	[-0.094,0.157]
Occupation								
Managerial/professional position	Reference		Reference		Reference		Reference	
Manual/service/part-time worker	0.974	[0.766,1.238]	0.956	[0.748,1.222]	0.070	[-0.031,0.172]	0.086	[-0.015,0.187]
Other	1.133	[0.836,1.536]	1.165	[0.852,1.593]	0.095	[-0.035,0.225]	0.113	[-0.016,0.242]
Party membership								
Party member	Reference		Reference		Reference		Reference	
Non-party member	1.028	[0.792,1.335]	1.044	[0.799,1.363]	0.011	[-0.100,0.121]	-0.004	[-0.114,0.106]
Monthly income								

No income	Reference		Reference		Reference		Reference	
≤2000	1.280	[0.950,1.725]	1.136	[0.837,1.542]	-0.071	[-0.197,0.055]	-0.064	[-0.189,0.062]
2001-4000	1.129	[0.851,1.499]	0.985	[0.737,1.316]	0.163**	[0.045,0.281]	0.184**	[0.067,0.302]
4001-6000	1.022	[0.743,1.406]	0.921	[0.664,1.277]	0.179**	[0.047,0.311]	0.186**	[0.055,0.318]
6001-8000	1.313	[0.878,1.963]	1.282	[0.850,1.931]	0.069	[-0.103,0.241]	0.069	[-0.102,0.241]
>8001	1.783*	[1.132,2.808]	1.480	[0.930,2.355]	0.312**	[0.113,0.511]	0.326**	[0.128,0.524]
Area								
Rural	Reference		Reference		Reference		Reference	
Urban	0.914	[0.648,1.291]	0.762	[0.535,1.084]	0.086	[-0.061,0.234]	0.103	[-0.045,0.251]
City								
Non-Wuhan	Reference		Reference		Reference		Reference	
Wuhan	0.912	[0.734,1.132]	0.822	[0.658,1.027]	-0.124**	[-0.215,-0.033]	-0.111*	[-0.201,-0.020]
Constant	0.316	[0.158,0.632]	3.393	[1.461,7.880]	3.344***	[3.051,3.637]	2.684***	[2.330,3.038]
Log lik.	-1319.53		-1273.05		-2851.45		-2837.18	

* $p < 0.05$, ** $p < 0.01$, *** $p < 0.001$.

Effects of changes in physical and sedentary behaviors on mental health and life satisfaction during the COVID-19 pandemic ~~lockdown~~: Evidence from China

Xi Chen[1*]

Haiyan Gao[2]

Binbin Shu[3]

Yuchun Zou[2]

[1] The Jockey Club School of Public Health and Primary Care, The Chinese University of Hong Kong

[2] Faculty of Humanities and Social Sciences, Beijing University of Technology

[3] Department of Sociology, Shenzhen University, China

[4] Institute of Sociology, Chinese Academy of Social Sciences, China

***Author for correspondence:**

Xi Chen

Research Assistant Professor

The Jockey Club School of Public Health and Primary Care

The Chinese University of Hong Kong

Email: celiaxichen@cuhk.edu.hk (XC)

Address: Room 419, School of Public Health Building, Prince of Wales Hospital, Shatin, Hong Kong

Abstract

Background: While restriction measures are critical in containing the COVID-19 outbreak, limited studies have investigated the behavioral and psychological impact of these measures. This study aimed to investigate the effects of physical and sedentary behavioral changes and online behavior during the COVID-19 pandemic on mental health and life satisfaction among the Chinese population.

Methods: The data were obtained from a cross-sectional survey of 2145 residents aged between 18 and 80 in Hubei province, China, whose capital city Wuhan first suffered from the COVID-19 pandemic between March 23, 2020, and April 9, 2020.

Results: Participants who had high frequencies of physical activities before or during the COVID-19 outbreak exhibited higher levels of life satisfaction. Participants who increased their sitting time during the pandemic or kept sitting for more than eight hours before and during the pandemic reported worse mental health than those who maintained less sedentary behavior. Besides, participants who used the Internet for information seeking, communication, and entertainment more frequently reported better mental health and life satisfaction. In contrast, there was a positive association between commercial use of the Internet and symptoms of mental disorders

Conclusion: Given the link between physical and sedentary behavioral changes with worse mental wellbeing, strategies to reduce sedentariness and increase physical activity during the COVID-19 pandemic are needed.

Keywords: physical activity, sedentary behavior, mental health, life satisfaction, COVID-19 pandemic, China

Introduction

Mental health during the COVID-19 pandemic among the general population in China

The COVID-19 pandemic has heavily impacted many societies and economies. The total confirmed infection cases had reached over 483 million, and the death toll hit more than 6.1 million by March 30, 2022.[1] To control the spreading of COVID-19, governments worldwide have implemented various restriction measures ranging from social distancing to societal shutdown.[2] While these measures are critical in containing the outbreak, they may compromise individuals' mental health and health activities.[2] [3]

The outbreak of COVID-19 was first reported in Wuhan, the capital city of Hubei province, China, in December 2019. Authorities have put Wuhan and 16 neighboring cities in Hubei under lockdown since January 23, 2020. During the following weeks, COVID-19 cases increased rapidly and overwhelmed the health care system, especially in Wuhan. The situation improved when the Chinese government built massive temporary medical facilities to house COVID patients and transferred a large number of medical personnel to Hubei. The lockdowns were relaxed since March 23, 2020 and on April 8, 2020, the lockdown in Wuhan (the hardest-hit city in China) was officially lifted. Given the unprecedented social distancing measures, numerous studies have reported a high prevalence of mental health problems among Chinese adults during the COVID-19 pandemic.[4] [5] [6] However, most prior studies focused on sociodemographic backgrounds, risk perceptions, and attitudes toward COVID-19 as risk factors for psychological distress.[7] [8] Limited studies have investigated the effect of pandemic-related behavioral changes on population mental health.

Mental health consequences of physical activity, sedentary behavior, and online activities

Numerous studies have shown that the COVID-19-related restriction measures disrupted daily routines and impacted lifestyle activities, including participation in sports and physical activity (PA) [9] [10]. The decline in PA was accompanied by increased sedentary (sitting) behavior [11]. Given the consistent benefits of regular PA for mental health [12] [13] [14], reductions in PA are likely to exacerbate COVID-19-fueled psychological distress. ~~A study of the general population in the UK revealed a negative association between moderate-to-vigorous PA (MVPA) and depressive or anxious symptoms during the COVID-19 pandemic [15]. Similar findings were found among adults in the US [16], Austria [17], and Italy [18]. Thus, individuals who keep themselves physically active during the COVID-19 pandemic might experience less mental distress and higher quality of life.~~ A review of 21 studies about the effect of PA on mental health during the CCOVID-19 pandemic suggested that people who performed PA regularly with high volume and frequency and kept the PA routines stable would experience fewer symptoms of anxiety and depression [15].

Besides, accumulating evidence has revealed that, independent of PA levels, sedentary behavior is associated with mental health [1916] [2017]. Several longitudinal investigations have shown that self-reported sedentary time predicts future mental health and wellbeing [2118] [2219]. A longitudinal study of populations in France and Switzerland found that sedentary behavior increased during the lockdown period of COVID-19, which was associated with poorer physical health, mental health, and subjective vitality [2320]. Increasing sitting time during the COVID-19 pandemic may be a risk factor for mental health and life satisfaction.

Moreover, COVID-19-related measures such as closing down schools, workplaces, and entertainment outlets coupled with self-quarantine have led to an unprecedented level of Internet and digital technology use. People move almost every aspect of their daily activities to cyberspace at an accelerating speed, as a “new normal” in the COVID-19 era. However, it remains unclear whether and

how Internet usage would exacerbate or alleviate COVID-19-fueled psychological distress. On the one hand, the Internet could enhance mental health by providing access to the latest information and enabling people to work, study, and shop online that regularizes their routines during quarantines. On the other hand, excessive engagement in specific online activities such as gambling, gaming, social media use, and shopping may result in severe problems and elevate the risk of disordered or addictive use of the Internet [2421] [2522]. Given the mixed evidence regarding the effects of Internet usage on mental health, more studies are warranted to investigate the impact of Internet usage on mental wellbeing during the COVID-19 pandemic.

To date, limited studies have examined the associations between changes in PA, sedentary behavior, and online behavior with mental health and quality of life among the Chinese population during the COVID-19 pandemic. A study of 4,898 adolescents in China showed that higher PA levels were significantly related to lower negative mood scores and higher positive mood scores during the COVID-19 pandemic [2623]. More engagement in physical activities was also associated with reduced anxiety and depression among Chinese college students after adjusting for confounding demographic factors [2724,25]. While providing important insights, these studies often focused on subpopulations (e.g., adolescents, college students), and thus their findings cannot be generalized to the general population. A study of the Chinese adult population found that compared with that before the lockdown, their time engaged in daily PA decreased and sedentary time increased, and PA is positively associated with life satisfaction [2826]. However, the authors did not examine the effect of COVID-19-induced behavioral changes on adverse mental health outcomes. Although some studies have found that PA participation was positively associated with mental health among the Chinese adults during the COVID-19 pandemic [27,28], they did not specifically examine how the changes in PA and sitting time before and during the COVID-19 pandemic may affect mental health. Moreover, virtually no research has investigated how different types of online behavior during the pandemic may affect the Chinese population's psychological wellbeing during the COVID-19 pandemic.

The present study

Given the lack of research on the effects of COVID-19-induced behavioral changes on mental wellbeing among the Chinese population, this study aims to investigate the impacts of behavioral changes in PA, sedentary behavior, and online behavior on individual psychological wellbeing and life satisfaction among residents in Hubei province, China, the original epicenter of the pandemic. The data collection was carried out between March 23, 2020, and April 9, 2020, while Hubei's lockdowns were gradually eased. Such data creates a unique opportunity for us to investigate Hubei residents' behavioral changes and psychological wellbeing during the pandemic. The findings of this study may improve our understanding and inform public health policies regarding the pattern and consequences of behavioral changes during the COVID-19 pandemic. Based on previous literature, we hypothesized that (1) the COVID-19 pandemic may be associated with changes in PA and sedentary behavior; (2) a decrease in PA may be negatively associated with mental health and life satisfaction; (3) an increase in sedentary behavior may have a negative effect on mental health and life satisfaction; and (4) Internet use may be positively associated with mental wellbeing and life satisfaction during the COVID-19 pandemic. In this study, we examined the impact of different types of Internet use, including information seeking, work/study, buying products, entertainment, reading/online learning, communication, and investment/financial management. ~~Given the mixed evidence about online behavior and mental health, we did not propose a specific hypothesis regarding the effect of Internet use on mental wellbeing and life satisfaction during the COVID-19 pandemic.~~

Methods

Data

The data came from the "Public Attitude toward The Novel Coronavirus Epidemic in Hubei Province" survey, which was carried out by the China Academy of Science and Technology Development Strategy, the Social Policy Research Institute at Renmin University, and the Institute of Sociology of the Chinese

Academy of Social Sciences. The survey was conducted between March 23, 2020, and April 9, 2020. Authorities imposed a strict lockdown policy for cities in Hubei since January 23, 2020, and started to ease the lockdown since March 23, 2020, with the lockdown in Wuhan officially lifted on April 8, 2020. ~~The data were thus suitable to investigate Hubei residents' behavioral changes and their associations with mental health and life satisfaction during the lockdown period.~~

The survey targeted all the residents aged between 18 and 80 in the urban and rural areas of Hubei. It was a combined online and phone survey. The online survey was conducted on a professional survey platform in China. The platform sent a notification to respondents in their sample bank with a link to allow access to the questionnaire. Only Hubei residents (with IP address locations in Hubei) can answer the questionnaire. No incentives were provided to the participants. A supplemented phone survey was carried out by trained research assistants to reach populations with limited access to the Internet (e.g., aged over 65, rural population). There are minimal differences between the two modes of data collection. Our research staff will be asked not to provide any additional information but simply read the questions to the participants. Participants who did the online survey provided written consent. Participants who answered the questions via phone gave oral consent, and the interviewer signed a form pledging that he/she had gone through the proper procedures to obtain the verbal informed consent of the participant. The study was approved by the ethical committee of the Chinese Academy of Social Science, and all procedures were in accordance with the 1964 Helsinki declaration and its later amendments or comparable ethical standards. In total, 2355 participants completed the online or phone surveys. After deleting cases with at least one missing value, the final sample included 2145 respondents, of which 1944 (90.63%) completed the online survey, and 201 (9.37%) completed the phone survey.

Measurements

Symptoms of mental disorders were measured by the 12-item Chinese Health Questionnaire scale (CHQ-12), which was widely used for screening non-psychotic mental health problems in the general population. Developed by Cheng and Williams (1986), CHQ was adapted from the General Health

Questionnaire [29] with the inclusion of culturally relevant items in Chinese societies. CHQ-12 has been validated in the general population in mainland China [30] [31], Taiwan [32] [33], and Chinese people in foreign countries [34] [35]. Ten items of CHQ-12 were negatively worded (e.g., "Been suffering from headache or pressure in your head") and two items were positively worded (e.g., "Have you been getting along well with your family or friends recently?"). The responses for all items ranged from 0 = "not at all" to 3 = "very often." The responses for positive ones were reversely coded so that higher values indicated higher levels of mental disorder. The internal validity of the scale was good (Chronbach's alpha = 0.78). In this study, the cut-off point against the diagnosis of non-psychotic disorders was 13/14. About 32.07% of the participants scored greater than or equal to 14 and could be regarded as having symptoms consistent with potential mental disorders.

Life satisfaction was assessed by the question, "In general, how satisfied are you with your life?" with a 5-point scale from 1 = "very dissatisfied" to 5 = "very satisfied." Many studies and large-scale surveys have used such a single-item life satisfaction measure [36] [37]. Prior research has confirmed the reliability of this single-item measure of life satisfaction by showing that it performed very similarly compared to the multiple-item Satisfaction with Life Scale (SWLS) and correlated with external variables (e.g., health and affect) to a similar degree [38] [39].

Physical activity. Participants reported the frequency of doing moderate-to-vigorous physical activity (MVPA) in 2019 and in the past month, followed by a brief explanation and some examples of MVPA. MVPA refers to exercises such as running, playing ball, swimming, dancing, brisk walking, etc., for ≥10 minutes, which cause breathing and heart rate to increase. The response categories ranged from "never" to "every day." Exercise training guidelines for a quarantine situation due to COVID-19 suggested that healthy or asymptomatic persons should do aerobic exercises for at least three sessions per week (four or five seem optimal) [41]. Participants were categorized as "maintaining high PA" if they did PA > two times a week, as "decreased PA" if they did PA for > two times a week before the COVID-19 pandemic but did not do so afterward, as "increased PA" if they increased PA to > two times

a week during the pandemic, and as "maintaining low PA" if they did PA for less than two times a week before and during the pandemic.

Sitting time. Participants reported average daily time spent sitting before (i.e., in early January 2020) and during the COVID-19 pandemic. Previous studies suggested that the association of daily sitting time and most long-term health outcomes is not linear, with a weekday sitting time below 8 hours/day associated with better perceived mental health and quality of life [40]. Participants were then classified as "maintaining low sitting time" if they reported ≤8h/day of sitting at both time points, as "increasing sitting time" if they reported ≤ 8h/day of sitting before the pandemic but reported > 8h/day of sitting after the pandemic, as "decreasing sitting time" if they reported > 8h/day of sitting before the pandemic but reported ≤ 8h/day of sitting after the pandemic, and as "maintaining high sitting time" if they reported > 8h/day of sitting at both time points.

Online behavior. Seven main and conceptually different types of Internet uses were accessed, including general information seeking; work; buying products (e.g., groceries); entertainment (e.g., playing online games, watching videos, listening to music); reading books/online class; communication with family and friends via online communication tools (e.g., WeChat); and investment and financial management. The responses ranged from 0 = "never" to 4 = "everyday". Higher values indicated a higher frequency of using the Internet for the corresponding activity.

In addition, a series of sociodemographic variables were measured, including age (continuous), sex (male vs. female), education (middle school or below, high school, college or above), occupation (managerial/professional position, manual/service/part-time worker, or other), China Communist Party membership (Party member vs. non-Party member), monthly personal income (from no income to >8000 *yuan*), area (rural vs. urban), city (Wuhan vs. non-Wuhan).

Analysis

All the analyses were performed using Stata 16.0. Descriptive statistics were used to characterize the study population. Considering the variable of potential mental disorder was binary, we fit multiple logistic regression models to study the associations between changes in sitting time and PA during the COVID-19 pandemic and online behavior with symptoms of potential mental disorders. For the outcome variable of life satisfaction, we used OLS regression models to examine the effect of sedentary activity, PA, and online behavior after adjusting for sociodemographic variables. Variance inflation factors (VIFs) of the independent variables were estimated to check whether multicollinearity exists in the models. All the VIFs were below 2, suggesting that multicollinearity was not a significant concern.

Results

~~Descriptive statistics~~

Table 1 displays the background characteristics of the respondents. ~~Of the 2145 respondents, the mean age was 33.70 (SD = 12.18), and about half of the respondents were female (51.28%). A majority of respondents were manual or service workers (59.77%), with a college education or above (65.50%), and with a monthly income of over 2000 *yuan* (60.79%). Most respondents lived in urban areas (92.30%), were non-Wuhan residents (74.78%), and were not Communist Party members (84.01%).~~

<Table 1 about here>

Table 2 shows the descriptive statistics of independent and dependent variables. ~~About 32.07% of participants reported symptoms of potential mental disorders. The mean score of general life satisfaction was 3.89 (out of a maximum of 5).~~ Compared to the pre-COVID-19 period, about 4.66% of participants increased their sitting time, and approximately 15.10% reduced their PA during the pandemic. As for various online behavior ~~rated on a 0–4 scale~~, participants were most frequently to use the Internet for information seeking ~~(M = 3.55)~~, followed by communication ~~(M = 3.36)~~, entertainment ~~(M = 3.33)~~,

reading, and attending the online class (M = 3.04), work and study (M = 2.91). Relatively fewer participants used the Internet for commercial activities, including buying products (M = 2.60) and investment and financial management (M = 1.95).

<Table 2 about here>

Regression results

Table 3 presents the associations between physical activities and sedentary behavior changes, and various online behavior with depression (Models 1 and 2) and life satisfaction (Models 3 and 4) after adjusting for sociodemographic variables. As shown in Model 1, compared to participants who sit less than 8 hours before and after the pandemic, those who increased their sitting time during the pandemic (OR = 2.384, CI = [1.579,3.600]) and kept sitting for more than 8 hours a day at both time points (OR = 2.022, CI = [1.229,3.324]) were more likely to experience symptoms of mental disorders. However, changes in the frequency of PA were not associated with symptoms of mental disorders. Model 2 examined the impact of digital engagement and revealed that information seeking (OR = 0.666, CI = [0.591,0.752]), entertainment (OR = 0.878, CI = [0.784,0.984]), and communication activities (OR = 0.857, CI = [0.766,0.958]) were associated with lower levels of symptoms of mental disorders. Conversely, online commercial activities, including buying products (OR = 1.217, CI = [1.093,1.354]) and doing investment online (OR = 1.181, CI = [1.091,1.277]) were positively associated with symptoms of mental disorders. Compared to those who participated in lower levels of physical activity, participants who increased (b = 0.246, CI = [0.040,0.453]), decreased (b = 0.238, CI = [0.081,0.395]), or maintained high frequencies of physical activity (b = 0.425, CI = [0.294,0.555]) exhibited higher levels of life satisfaction. In contrast to the mixed effect of digital engagement on depression, most online behaviors positively effect on life satisfaction. Specifically, using the Internet for information seeking (b = 0.095, CI = [0.043,0.148]), study/work (b = 0.052, CI = [0.018,0.086]), reading books/online class (b = 0.054, CI = [0.018,0.091]), and communication (b = 0.048, CI = [0.001,0.096]) were positively associated with life satisfaction. Other online behaviors, such as buying products,

entertainment, and investment/financial management, were not significantly associated with life satisfaction.

<Table 3 about here>

Discussion

This study presents a timely investigation of the effects of behavioral changes (i.e., PA, sedentary behavior, and online behavior) induced by COVID-19-related restrictions on psychological wellbeing and life satisfaction among the general population in Hubei province, China, the original epicenter of the pandemic. With data obtained from 2145 Hubei residents during the pandemic, the findings showed (1) an expected decline in PA and an increase in sitting time during the pandemic; (2) an association between increased sitting time and symptoms of mental disorder; (3) higher life satisfaction for those doing more exercises; and (4) a mixed pattern of Internet uses and mental health and life satisfaction.

While it is well-documented that increased PA could reduce mental health problems [42] [43] [44], there is insufficient evidence regarding the effect of PA on positive mental health among the Chinese population. The findings of our study revealed that more involvement in PA during the COVID-19 pandemic might effectively improve life satisfaction among Hubei residents. PA could be associated with life satisfaction through fitness- and health-related adaptations that enhance physical and mental health. PA may also have a revitalizing effect that strengthens self-control and supports goal pursuits [45] [46]. Considering we live in times of great psychological and emotional fragility, it is even more essential to promote PA that may help people adapt to the crisis and gain more life satisfaction. Our findings suggest that those who have low frequencies of PA during COVID-19 social distancing may need to be targeted with support for their quality of life. Policymakers and stakeholders should develop health education and communication that emphasize PA to improve life satisfaction during an infectious disease outbreak. As people have limited opportunities for doing PA outside their home during social distancing, future PA intervention to foster an Active and Healthy Confinement Lifestyle

(AHCL) during a pandemic may adopt ICT solutions, such as home-based exercise games and fitness apps.

Besides, our findings indicated that the overall time spent in sedentary behavior is positively associated with symptoms of mental disorders. Sedentary behavior could be associated with mental disorders via several possible pathways. For example, screen-based sedentary behaviors (e.g., TV viewing) are likely to induce addiction [47], internalizing symptoms [48], and low sleeping quality [49] , potentially leading to heightened levels of mental distress. Further, engaging in sedentary behaviors may displace time spent in other activities such as household or work-related responsibilities or PA, which may be effective coping strategies for symptoms of mental disorders [50]. It is worth noting that specific sedentary behaviors may contribute to mental health in different ways. For example, a study found that specific screen-based recreation was associated with higher self-esteem among Australian adolescents in low-income communities [51]. Thus, future studies may investigate associations between different types of sedentary behavior and mental health.

Our findings showed that various Internet use types had different effects on mental health and life satisfaction during the COVID-19 pandemic among Hubei residents. To start with, online information-seeking behavior was associated with a lower likelihood of mental disorders and increased life satisfaction. Such findings seemed inconsistent with other studies showing that exposure to COVID-19-related information may increase psychological distress [52] [53]. The discrepancy between our study and others may be that we measured the general information-seeking behavior rather than COVID-19 specific information seeking. Future studies may further investigate whether information-seeking behavior in general and about COVID-19 has differential effects on psychological wellbeing.

Also, online communication activity played an important role in improving mental health and life satisfaction, which appeared contradictory to prior studies showing that online communication (especially the use of social media) may deteriorate mental health due to its negative impact on face-to-face communications [54] [55]. Considering offline interactions were restricted by physical distancing

and stay-at-home orders during the COVID-19 pandemic, online contact with friends and family may increase emotional support, decrease loneliness and social isolation, which leads to lower depression and improved life satisfaction. Besides, our results have shown that online entertainment can reduce symptoms consistent with potential mental disorders. Using the Internet to relax may be a positive coping strategy to alleviate feelings of stress and isolation [56]. Similarly, working and learning remotely during the pandemic may keep people connected with their co-workers and classmates, despite physical isolation, and thus were positively associated with life satisfaction. Using the Internet for work and study may also help regularize daily routines, which is essential for buffering the adverse impact of stress exposure during a crisis [57].

While respondents who used the Internet for information seeking, communication, study/work, reading books, and entertainment reported better mental health and life satisfaction, the commercial use of the Internet (e.g., shopping, investment) seemed to increase mental distress. We conjecture that the economic downturn and uncertainty caused by the pandemic may be why people who were involved in more online economic activities suffered more distress. Previous studies also found that online shopping may be a negative coping strategy for stress and was associated with increased symptoms of mental disorders [58] [59]. While online purchases were the dominant way for people to get groceries due to social distancing and stay-at-home orders, scholars have suggested setting a specific time and financial limits for online shopping to reduce the risk of disordered or addictive use [60].

Despite the significant findings, this study is not without limitations. First, due to the cross-sectional design employed in the present study, it cannot ascertain whether mental health problems lead to lower PA levels or whether lower levels of PA lead to mental health problems during the COVID-19 pandemic. Additional longitudinal studies are needed to infer the direction of the association. Second, our samples are not representative since it was mainly conducted online. However, considering in-person surveys would not be appropriate or possible during the COVID-19 pandemic, online surveys are arguably the most feasible way to collect data in a timely matter. We have also supplemented the online survey with a telephone survey to reach groups with limited access to the Internet. Future studies may try various

sampling methods to gain a population-based representative sample. Third, participants were asked to self-report their PA level, thus introducing self-reporting bias into the findings. Moreover, we asked participants to retrospectively report their physical activity and sitting time prior to the COVID-19 pandemic, which may be subject to recall bias due to inaccurate memory. Future studies may use objective or direct measures of physical activity to increase precision and validate the self-report measures. Lastly, due to the online survey's length limitation, we only measured the PA frequency and the total amount of sedentary behavior during the pandemic. Future studies may further specify the level and type of PA and sedentary behavior and their relationship with mental health and life satisfaction.

Conclusion

In conclusion, social distancing and quarantine measures to mitigate the COVID-19 pandemic may affect PA, mental health, and life satisfaction among the general population, with those experiencing a decrease in PA having lower life satisfaction, and those sitting more time reporting a higher likelihood of mental disorders. Besides, the increasing use of the Internet and digital technology throughout the pandemic also impacts individuals' mental health and life satisfaction. Our findings showed that participants who used the Internet for information seeking, communication, study/work, reading books, and entertainment more frequently reported better mental health and life satisfaction. In contrast, the commercial use of the Internet (e.g., shopping, investment) seemed to increase mental distress.

Acknowledgment

This work was supported by the National Social Science Fund of China (grant no. 16ZDA079). The funders had no role in study design, data collection and analysis, decision to publish, or preparation of the manuscript.

References:

[1] World Health Organization. WHO Coronavirus (COVID-19) Dashboard. Available at: https://covid19.who.int/ (accessed on March 30, 2022)

[2] Benke C, Autenrieth LK, Asselmann E, Pané-Farré CA. Lockdown, quarantine measures, and social distancing: Associations with depression, anxiety and distress at the beginning of the COVID-19 pandemic among adults from Germany. Psychiatry Research. 2020 Nov 1;293:113462.

[3] Brooks SK, Webster RK, Smith LE, Woodland L, Wessely S, Greenberg N, Rubin GJ. The psychological impact of quarantine and how to reduce it: rapid review of the evidence. The Lancet. 2020 Feb 26.

[4] Qiu J, Shen B, Zhao M, Wang Z, Xie B, Xu Y. A nationwide survey of psychological distress among Chinese people in the COVID-19 epidemic: implications and policy recommendations. General psychiatry. 2020;33(2).

[5] Tang W, Hu T, Hu B, Jin C, Wang G, Xie C, Chen S, Xu J. Prevalence and correlates of PTSD and depressive symptoms one month after the outbreak of the COVID-19 epidemic in a sample of home-quarantined Chinese university students. Journal of affective disorders. 2020 May 13.

[6] Xin M, Luo S, She R, Yu Y, Li L, Wang S, Ma L, Tao F, Zhang J, Zhao J, Li L. Negative cognitive and psychological correlates of mandatory quarantine during the initial COVID-19 outbreak in China. American Psychologist. 2020 Jul;75(5):607.

[7] Kwok KO, Li KK, Chan HH, Yi YY, Tang A, Wei WI, Wong YS. Community responses during the early phase of the COVID-19 epidemic in Hong Kong: risk perception, information exposure and preventive measures. MedRxiv. 2020 Jan 1.

[8] Xiong J, Lipsitz O, Nasri F, Lui LM, Gill H, Phan L, Chen-Li D, Iacobucci M, Ho R, Majeed A, McIntyre RS. Impact of COVID-19 pandemic on mental health in the general population: A systematic review. Journal of affective disorders. 2020 Aug 8.

[9] Ammar A, Brach M, Trabelsi K, Chtourou H, Boukhris O, Masmoudi L, Bouaziz B, Bentlage E, How D, Ahmed M. Effects of COVID-19 home confinement on social participation and life satisfaction: Preliminary results of the ECLB-COVID19 international online-survey. medRxiv. 2020.

[10] Barwais FA, Cuddihy TF, Tomson LM. Physical activity, sedentary behavior and total wellness changes among sedentary adults: a 4-week randomized controlled trial. Health and quality of life outcomes. 2013 Dec 1;11(1):183.

[11] Hall G, Laddu DR, Phillips SA, Lavie CJ, Arena R. A tale of two pandemics: How will COVID-19 and global trends in physical inactivity and sedentary behavior affect one another?. Progress in Cardiovascular Diseases. 2020 Apr 8.

[12] Taylor CB, Sallis JF, Needle R. The relation of physical activity and exercise to mental health. Public health reports. 1985 Mar;100(2):195.

[13] Deslandes A, Moraes H, Ferreira C, Veiga H, Silveira H, Mouta R, Pompeu FA, Coutinho ES, Laks J. Exercise and mental health: many reasons to move. Neuropsychobiology. 2009;59(4):191-8.

[14] Ashdown-Franks G, Firth J, Carney R, Carvalho AF, Hallgren M, Koyanagi A, Rosenbaum S, Schuch FB, Smith L, Solmi M, Vancampfort D. Exercise as medicine for mental and substance use disorders: a meta-review of the benefits for neuropsychiatric and cognitive outcomes. Sports Medicine. 2020 Jan 1:1-20.

[15] Jacob L, Tully MA, Barnett Y, Lopez-Sanchez GF, Butler L, Schuch F, López-Bueno R, McDermott D, Firth J, Grabovac I, Yakkundi A. The relationship between physical activity and mental health in a sample of the UK public: A cross-sectional study during the implementation of COVID-19 social distancing measures. Mental Health and Physical Activity. 2020 Oct 1;19:100345.

[16] Meyer J, McDowell C, Lansing J, Brower C, Smith L, Tully M, Herring M. Changes in physical activity and sedentary behavior in response to COVID-19 and their associations with mental health in 3052 US adults. International journal of environmental research and public health. 2020 Sep;17(18).

[17] Pieh C, Budimir S, Probst T. The effect of age, gender, income, work, and physical activity on mental health during coronavirus disease (COVID-19) lockdown in Austria. Journal of psychosomatic research. 2020 Sep 1;136:110186.

[18] Maugeri G, Castrogiovanni P, Battaglia G, Pippi R, D'Agata V, Palma A, Di Rosa M, Musumeci G. The impact of physical activity on psychological health during Covid-19 pandemic in Italy. Heliyon. 2020 Jun 1;6(6):e04315.

[15] Wolf S, Seiffer B, Zeibig JM, Welkerling J, Brokmeier L, Atrott B, Ehring T, Schuch FB. Is physical activity associated with less depression and anxiety during the COVID-19 pandemic? A rapid systematic review. Sports Medicine. 2021 Aug;51(8):1771-83.

[16] Salmon P. Effects of physical exercise on anxiety, depression, and sensitivity to stress: a unifying theory. Clinical psychology review. 2001 Feb 1;21(1):33-61.

[17] Wilcox S, Dowda M, Leviton LC, Bartlett-Prescott J, Bazzarre T, Campbell-Voytal K, Carpenter RA, Castro CM, Dowdy D, Dunn AL, Griffin SF. Active for life: final results from the translation of two physical activity programs. American journal of preventive medicine. 2008 Oct 1;35(4):340-51.

[18] Lucas M, Mekary R, Pan A, Mirzaei F, O'Reilly ÉJ, Willett WC, Koenen K, Okereke OI, Ascherio A. Relation between clinical depression risk and physical activity and time spent watching television in older women: a 10-year prospective follow-up study. American journal of epidemiology. 2011 Nov 1;174(9):1017-27.

[19] Sanchez-Villegas A, Ara I, Guillen-Grima F, Bes-Rastrollo M, Varo-Cenarruzabeitia JJ, Martinez-Gonzalez MA. Physical activity, sedentary index, and mental disorders in the SUN cohort study. Medicine & Science in Sports & Exercise. 2008 May 1;40(5):827-34.

[20] Cheval B, Sivaramakrishnan H, Maltagliati S, Fessler L, Forestier C, Sarrazin P, Orsholits D, Chalabaev A, Sander D, Ntoumanis N, Boisgontier MP. Relationships between changes in self-reported physical activity, sedentary behaviour and health during the coronavirus (COVID-19) pandemic in France and Switzerland. Journal of sports sciences. 2020 Oct 31:1-6.

[21] Fineberg NA, Apergis-Schoute AM, Vaghi MM, Banca P, Gillan CM, Voon V, Chamberlain SR, Cinosi E, Reid J, Shahper S, Bullmore ET. Mapping compulsivity in the DSM-5 obsessive compulsive and related disorders: cognitive domains, neural circuitry, and treatment. International Journal of Neuropsychopharmacology. 2018 Jan;21(1):42-58.

[22] Peeters M, Koning I, Lemmens J, Eijnden RV. Normative, passionate, or problematic? Identification of adolescent gamer subtypes over time. Journal of behavioral addictions. 2019 Sep;8(3):574-85.

[23] Kang S, Sun Y, Zhang X, Sun F, Wang B, Zhu W. Is Physical Activity Associated with Mental Health among Chinese Adolescents during Isolation in COVID-19 Pandemic?. Journal of Epidemiology and Global Health. 2020 Sep.

[24] Xiang MQ, Tan XM, Sun J, Yang HY, Zhao XP, Liu L, Hou XH, Hu M. Relationship of physical activity with anxiety and depression symptoms in Chinese college students during the COVID-19 outbreak. Frontiers in Psychology. 2020;11.

[25] Zhang Y, Wu X, Tao S, Li S, Ma L, Yu Y, Sun G, Li T, Tao F. Associations between screen time, physical activity, and depressive symptoms during the 2019 coronavirus disease (COVID-19) outbreak among Chinese college students. Environmental Health and Preventive Medicine. 2021 Dec;26(1):1-2.

[26] Wang X, Lei SM, Le S, Yang Y, Zhang B, Yao W, Gao Z, Cheng S. Bidirectional influence of the COVID-19 pandemic lockdowns on health behaviors and quality of life among Chinese adults. International Journal of Environmental Research and Public Health. 2020 Jan;17(15):5575.

[27] Qi M, Li P, Moyle W, Weeks B, Jones C. Physical activity, health-related quality of life, and stress among the Chinese adult population during the COVID-19 pandemic. International journal of environmental research and public health. 2020 Jan;17(18):6494.

[28] Nie Y, Ma Y, Wu Y, Li J, Liu T, Zhang C, Lv C, Zhu J. Association between physical exercise and mental health during the COVID-19 outbreak in China: a nationwide cross-sectional study. Frontiers in Psychiatry. 2021:1381.

[29] Goldberg DP. User's guide to the General Health Questionnaire. Windsor. 1988.

[30] Yang TZ, Huang L, Wu ZY. The application of Chinese health questionnaire for mental disorder screening in community settings in mainland China. Zhonghua Liu Xing Bing Xue Za Zhi= Zhonghua Liuxingbingxue Zazhi. 2003 Sep 1;24(9):769-73.

[31] Zhang CC, Hou LH, Zheng X, Lu J, Zou JY, Qian Y, Yang TZ. Factor structure of 12 items in the Chinese Health Questionnaire among the elderly population in mainland China. Public Health. 2020 Oct 1;187:143-9.

[32] Cheng TA, Williams P. The design and development of a screening questionnaire (CHQ) for use in community studies of mental disorders in Taiwan. Psychological medicine. 1986 May;16(2):415-22.

[33] Cheng TA, Wu JT, Chong MY, Williams P. Internal consistency and factor structure of the Chinese Health Questionnaire. Acta Psychiatrica Scandinavica. 1990 Oct;82(4):304-8.

[34] Pan PC, Goldberg DP. A comparison of the validity of GHQ-12 and CHQ-12 in Chinese primary care patients in Manchester. Psychological Medicine. 1990 Nov;20(4):931-40.

[35] Barbato A, Aresu A, Battino RN, Troisi E, Tettamanti M, Parabiaghi A. A tool for cross-cultural psychiatry research: a pilot study of adaptation of 12-item Chinese Health Questionnaire (CHQ-12) for Chinese population in Italy. Rivista di psichiatria. 2009;44(4):249-57.

[36] Wagner GG, Joachim RF, Jürgen S. The German Socio-Economic Panel Study (SOEP)-Scope, Evolution and Enhancements. Schmollers Jahrbuch: Journal of Applied Social Science Studies. 2007;127(1):139–169.

[37] Taylor MF, Brice J, Buck N, Prentice-Lane E. British Household Panel Survey user manual volume A: Introduction, technical report, and appendices. Colchester: University of Essex; 2009. Available at: http://iserwww.essex.ac.uk/ulsc/bhps/doc/

[38] Kobau R, Sniezek J, Zack MM, Lucas RE, Burns A. Well-Being Assessment: An Evaluation of Well-Being Scales for Public Health and Population Estimates of Well-Being among US Adults. Applied Psychology: Health and Well-Being. 2010;2(3):272–297.

[39] Cheung F, Lucas RE. Assessing the validity of single-item life satisfaction measures: Results from three large samples. Quality of Life research. 2014 Dec 1;23(10):2809-18.

[40] Gibson AM, Muggeridge DJ, Hughes AR, Kelly L, Kirk A. An examination of objectively-measured sedentary behavior and mental wellbeing in adults across week days and weekends. PloS one. 2017 Sep 21;12(9):e0185143.

[41] Khoramipour K, Basereh A, Hekmatikar AA, Castell L, Ruhee RT, Suzuki K. Physical activity and nutrition guidelines to help with the fight against COVID-19. Journal of Sports Sciences. 2020 Aug 27:1-7.

[42] McDowell CP, Dishman RK, Gordon BR, Herring MP. Physical activity and anxiety: a systematic review and meta-analysis of prospective cohort studies. American journal of preventive medicine. 2019 Oct 1;57(4):545-56.

[43] Schuch FB, Vancampfort D, Firth J, Rosenbaum S, Ward PB, Silva ES, Hallgren M, Ponce De Leon A, Dunn AL, Deslandes AC, Fleck MP. Physical activity and incident depression: a meta-analysis of prospective cohort studies. American Journal of Psychiatry. 2018 Jul 1;175(7):631-48.

[44] White RL, Babic MJ, Parker PD, Lubans DR, Astell-Burt T, Lonsdale C. Domain-specific physical activity and mental health: a meta-analysis. American journal of preventive medicine. 2017 May 1;52(5):653-66.

[45] Penedo FJ, Dahn JR. Exercise and wellbeing: a review of mental and physical health benefits associated with physical activity. Current opinion in psychiatry. 2005 Mar 1;18(2):189-93.

[46] Rejeski WJ, Mihalko SL. Physical activity and quality of life in older adults. The Journals of Gerontology Series A: Biological sciences and medical sciences. 2001 Oct 1;56(suppl_2):23-35.

[47] Cheng YS, Tseng PT, Lin PY, Chen TY, Stubbs B, Carvalho AF, Wu CK, Chen YW, Wu MK. Internet addiction and its relationship with suicidal behaviors: a meta-analysis of multinational observational studies. The Journal of clinical psychiatry. 2018 Jun 5;79(4):0-.

[48] Zink J, Belcher BR, Imm K, Leventhal AM. The relationship between screen-based sedentary behaviors and symptoms of depression and anxiety in youth: a systematic review of moderating variables. BMC public health. 2020 Dec;20:1-37.

[49]Fossum IN, Nordnes LT, Storemark SS, Bjorvatn B, Pallesen S. The association between use of electronic media in bed before going to sleep and insomnia symptoms, daytime sleepiness, morningness, and chronotype. Behavioral sleep medicine. 2014 Sep 3;12(5):343-57.

[50] Teychenne M, Hinkley T. Associations between screen-based sedentary behaviour and anxiety symptoms in mothers with young children. PLoS One. 2016 May 18;11(5):e0155696.

[51]Nihill GF, Lubans DR, Plotnikoff RC. Associations between sedentary behavior and self-esteem in adolescent girls from schools in low-income communities. Mental Health and Physical Activity. 2013;6(1):30-5.

[52] Chao M, Chen X, Liu T, Yang H, Hall BJ. Psychological distress and state boredom during the COVID-19 outbreak in China: the role of meaning in life and media use. European journal of psychotraumatology. 2020 Dec 31;11(1):1769379.

[53] Gao J, Zheng P, Jia Y, Chen H, Mao Y, Chen S, Wang Y, Fu H, Dai J. Mental health problems and social media exposure during COVID-19 outbreak. Plos one. 2020;15(4):e0231924.

[54] Caplan SE. Preference for online social interaction: A theory of problematic Internet use and psychosocial wellbeing. Communication research. 2003 Dec;30(6):625-48.

[55] Kim J, LaRose R, Peng W. Loneliness as the cause and the effect of problematic Internet use: The relationship between Internet use and psychological wellbeing. Cyberpsychology & behavior. 2009 Aug 1;12(4):451-5.

[56] Pressman SD, Matthews KA, Cohen S, Martire LM, Scheier M, Baum A, Schulz R. Association of enjoyable leisure activities with psychological and physical wellbeing. Psychosomatic medicine. 2009 Sep;71(7):725.

[57] Hou WK, Lai FT, Ben-Ezra M, Goodwin R. Regularizing daily routines for mental health during and after the COVID-19 pandemic. Journal of Global Health. 2020 Dec;10(2).

[58] Morgan C, Cotten SR. The relationship between Internet activities and depressive symptoms in a sample of college freshmen. CyberPsychology & Behavior. 2003 Apr 1;6(2):133-42.

[59]Norberg MM, Crone C, Kwok C, Grisham JR. Anxious attachment and excessive acquisition: The mediating roles of anthropomorphism and distress intolerance. Journal of Behavioral Addictions. 2018 Mar;7(1):171-80..

[60] Király O, Potenza MN, Stein DJ, King DL, Hodgins DC, Saunders JB, Griffiths MD, Gjoneska B, Billieux J, Brand M, Abbott MW. Preventing problematic internet use during the COVID-19 pandemic: Consensus guidance. Comprehensive Psychiatry. 2020 May 12:152180.

Table 1. Descriptive statistics of background variables

Variable	N (%)	
Age, Mean (SD)	33.70 (12.80)	
Sex		
Male	1045	(48.72)
Female	1100	(51.28)
Education		
Middle school or below	257	(11.98)
High school	483	(22.52)
College or above	1405	(65.50)
Occupation		
Managerial/professional position	495	(23.08)
Manual/service worker	1282	(59.77)
Other	368	(17.16)
Communist Party membership		
Party member	343	(15.99)
Non-Party member	1802	(84.01)
Monthly income		
No income	390	(18.18)
≤2000	451	(21.03)
2001-4000	642	(29.93)
4001-6000	394	(18.37)
6001-8000	159	(7.41)
>8001	108	(5.08)
Area		
Rural	165	(7.69)
Urban	1980	(92.31)
City		
Wuhan	541	(25.22)
Non-Wuhan	1604	(74.78)

Table 2. Descriptive statistics of dependent and independent variables

Variable	N (%)	Mean (SD)
Mental Health (CHQ-12)		
Probable mental disorders	688 (32.07)	
No probable mental disorders	1457 (67.93)	
Life satisfaction		3.89 (0.94)
Physical activity		
Maintained low	229 (10.68)	
Increased	117 (5.45)	
Decreased	324 (15.10)	
Maintained high	1475 (68.76)	
Sitting time		
Maintained low	1922 (89.60)	
Increased	100 (4.66)	
Decreased	53 (2.47)	
Maintained high	70 (3.26)	
Online behavior		
Information seeking		3.55 (0.80)
Work/study		2.91 (1.27)
Buying products		2.60 (1.06)
Entertainment		3.33 (0.91)
Reading/online learning		3.04 (1.17)
Communication		3.36 (0.94)
Investment/financial management		1.95 (1.35)

Table 3. Adjusted associations between self-reported changes in behavior before and during the COVID-19 pandemic and mental health and life satisfaction.

	Probable mental disorders				Life satisfaction			
	Model 1		Model 2		Model 3		Model 4	
	OR	95% CI	OR	95% CI	b	95% CI	b	95% CI
Physical activity								
Maintained low	Reference				Reference			
Increased	1.492	[0.924,2.409]			0.246*	[0.040,0.453]		
Decreased	0.721	[0.488,1.065]			0.238**	[0.081,0.395]		
Maintained high	1.322	[0.966,1.808]			0.425***	[0.294,0.555]		
Sitting time								
Maintained low	Reference				Reference			
Increased	2.384***	[1.579,3.600]			-0.010	[-0.197,0.176]		
Decreased	1.031	[0.564,1.884]			-0.015	[-0.268,0.237]		
Maintained high	2.022**	[1.229,3.324]			-0.188	[-0.411,0.035]		
Online behavior								
Information seeking			0.666***	[0.591,0.752]			0.095***	[0.043,0.148]
Work/study			0.940	[0.863,1.024]			0.052**	[0.018,0.086]
Buying products			1.217***	[1.093,1.354]			0.039	[-0.004,0.081]
Entertainment			0.878*	[0.784,0.984]			0.012	[-0.036,0.060]
Reading/online learning			0.953	[0.871,1.042]			0.054**	[0.018,0.091]
Communication			0.857**	[0.766,0.958]			0.048*	[0.001,0.096]
Investment/financial management			1.181***	[1.091,1.277]			-0.006	[-0.038,0.027]
Sex								
Male	Reference		Reference		Reference		Reference	
Female	0.958	[0.795,1.153]	0.981	[0.811,1.186]	-0.036	[-0.115,0.043]	-0.039	[-0.117,0.040]
Age	1.004	[0.996,1.011]	1.003	[0.995,1.011]	-0.001	[-0.004,0.003]	0.000	[-0.003,0.003]
Education								
Middle school or below	Reference		Reference		Reference		Reference	
High school	1.028	[0.737,1.434]	1.042	[0.741,1.464]	0.078	[-0.063,0.220]	0.077	[-0.063,0.218]
College or above	0.980	[0.728,1.319]	0.961	[0.709,1.302]	0.041	[-0.085,0.167]	0.031	[-0.094,0.157]
Occupation								
Managerial/professional position	Reference		Reference		Reference		Reference	
Manual/service/part-time worker	0.974	[0.766,1.238]	0.956	[0.748,1.222]	0.070	[-0.031,0.172]	0.086	[-0.015,0.187]
Other	1.133	[0.836,1.536]	1.165	[0.852,1.593]	0.095	[-0.035,0.225]	0.113	[-0.016,0.242]
Party membership								
Party member	Reference		Reference		Reference		Reference	
Non-party member	1.028	[0.792,1.335]	1.044	[0.799,1.363]	0.011	[-0.100,0.121]	-0.004	[-0.114,0.106]
Monthly income								

No income	Reference		Reference		Reference		Reference	
≤2000	1.280	[0.950,1.725]	1.136	[0.837,1.542]	-0.071	[-0.197,0.055]	-0.064	[-0.189,0.062]
2001-4000	1.129	[0.851,1.499]	0.985	[0.737,1.316]	0.163**	[0.045,0.281]	0.184**	[0.067,0.302]
4001-6000	1.022	[0.743,1.406]	0.921	[0.664,1.277]	0.179**	[0.047,0.311]	0.186**	[0.055,0.318]
6001-8000	1.313	[0.878,1.963]	1.282	[0.850,1.931]	0.069	[-0.103,0.241]	0.069	[-0.102,0.241]
>8001	1.783*	[1.132,2.808]	1.480	[0.930,2.355]	0.312**	[0.113,0.511]	0.326**	[0.128,0.524]
Area								
Rural	Reference		Reference		Reference		Reference	
Urban	0.914	[0.648,1.291]	0.762	[0.535,1.084]	0.086	[-0.061,0.234]	0.103	[-0.045,0.251]
City								
Non-Wuhan	Reference		Reference		Reference		Reference	
Wuhan	0.912	[0.734,1.132]	0.822	[0.658,1.027]	-0.124**	[-0.215,-0.033]	-0.111*	[-0.201,-0.020]
Constant	0.316	[0.158,0.632]	3.393	[1.461,7.880]	3.344***	[3.051,3.637]	2.684***	[2.330,3.038]
Log lik.	-1319.53		-1273.05		-2851.45		-2837.18	

* $p < 0.05$, ** $p < 0.01$, *** $p < 0.001$.

Response Letter

Editor's Comment

1.Please clarify the reasons for the restrictions on data availability. Stating "data available upon reasonable request from the corresponding author" is not sufficient. Please explain your exceptional situation.

Response: We have clarified the reasons for the restrictions on data availability, as shown below.

"Data cannot be shared publicly because of data protection regulation. Data are available from the Chinese Social Quality Data Archive for researchers who meet the criteria for access to confidential data. The data are available for research upon reasonable request and with permission from the Chinese Social Quality Data Archive: http://csqr.cass.cn/index.jsp"

2. This is a cross-sectional study using survey methodology. Your limitations section (pages 13-14) does a very good job of conveying this material about the cross-sectional nature of the design. As you note, this type of design does not allow statements about one variable causing a change in another variable. For this reason, statements such as "the commercial use of the internet seemed to increase mental disorders" (in the abstract) should be phrased as "there was an association between commercial use of the internet and symptoms of mental disorders." (Note comment 4 below that mental disorders per se were not assessed; rather, a self-report measure of symptoms of mental disorders was completed). Likewise, the statement "we hypothesized that (1) the lockdown may cause a decrease in PA and an increase in sedentary behavior" (page 6, as numbered) is not appropriate for this design. Please rewrite as "....the lockdown may be associated with changes in PA and sedentary behavior." More generally, throughout the writing, please edit to make sure the language used reflects the cross-sectional design and does not suggest causality or a longitudinal design.

Response: Thanks for your comment. We have revised the manuscript accordingly, using language that reflects the cross-sectional design. We detailed the changes below.

(1) We have rephrased the sentence "the commercial use of the internet seemed to increase mental disorders" (in the Abstract) as "there was a positive association between commercial use of the internet and symptoms of mental disorders."

(2) As the editor suggested, we have revised the first hypothesis as "the COVID-19 pandemic may be associated with changes in PA and sedentary behavior." (p.6)

(3) We have revised the second hypothesis, "a decrease in PA may deteriorate mental health and life satisfaction," as "a decrease in PA may be negatively associated with mental health and life satisfaction." (p.6)

3. Please clarify the consent process for the participants who went through a phone survey. Did participants who answered the questions via phone give oral consent, and those who did the online survey provide written consent?

Response: We have added information about the consent process in the revised manuscript (p.7).

"Participants who did the online survey provided written consent. Participants who answered the questions via phone gave oral consent, and the interviewer signed on a form pledging that he/she had gone through the proper procedures to obtain the verbal informed consent of the participant."

4. The self-report measure used does not permit a true diagnosis of mental disorders. I recommend using language that indicates that the measure reports on symptoms consistent with a potential mental disorder.

Response: Thanks for the comment. We have revised the manuscript using language that indicates that the measure reports on symptoms consistent with a potential mental disorder. For example,

(1) We rewrote the sentence "Mental health was measured by the 12-item Chinese Health Questionnaire scale (CHQ-12)" as "Symptoms of mental disorders were measured by the 12-item Chinese Health Questionnaire scale (CHQ-12)". (p.7)

(2) We have rephrased the sentence, "About 32.07% of the participants scored greater than or equal to 14 and could be regarded as having potential mental disorders." as "About 32.07% of the participants scored greater than or equal to 14 and could be regarded as having symptoms consistent with a potential mental disorder." (p.8)

(3) We have rephrased the sentence, "we fit multiple logistic regression models to study the associations between changes in sitting time and PA during the lockdown period of COVID-19 and online behavior with potential mental disorders." as "we fit multiple logistic regression models to study the associations between changes in sitting time and PA during the lockdown period of COVID-19 and online behavior with symptoms of potential mental disorders."

5. What is meant by depression, page 10 as numbered? I don't see a measure of depression. Is depression used as a synonym for "mental disorders"? Please harmonize the language. If the self-report measure is a measure of mental disorders, then the term mental disorders should be used to refer to findings from this measure.

Response: Thanks for the comment. We used depression as a synonym for mental disorders in the original manuscript. We have harmonized the language in the revised manuscript, particularly in the results and discussion parts. For example, we rewrote the sentence, "kept sitting for more than 8 hours a day at both time points were more likely to develop depressive symptoms" as "kept sitting for more than 8 hours a day at both time points were more likely to experience symptoms of mental disorders" (p.1q).

We rewrote the sentence, "However, changes in the frequency of PA were not associated with depression" as "However, changes in the frequency of PA were not associated with symptoms of mental disorders" (p.11). Besides, we revised the sentence, "Previous studies also found that online shopping may be a negative coping strategy for stress and was associated with increased depression" as "Previous studies also found that online shopping may be a negative coping strategy for stress and was associated with increased symptoms of mental disorders." (p.13)

6. Please make sure all the dates and details given in the text about the pandemic are correct. It is beyond the scope of the review process to verify this information.

Response: We have double-checked the dates and details about the pandemic and confirmed that the details are correct.

7. Please make sure that your literature review is comprehensive and includes recent research on physical activity during the pandemic, such as this systematic review:

Wolf, S., Seiffer, B., Zeibig, J., Welkerling, J., Brokmeier, L., Atrott, B., Ehring, T., & Schuch, F. B. (2021). Is physical activity associated with less depression and anxiety during the COVID-19 pandemic? A rapid systematic review. Sports Medicine (Auckland), 51(8), 1771-1783. [*https://doi.org/10.1007/s40279-021-01468-z*](https://doi.org/10.1007/s40279-021-01468-z)

Response: Thanks for the comment. We have conducted a more comprehensive literature review and added the reference suggested by the Editor and some more recent studies in China in the revised manuscript. The details of the changes are shown on pp. 4-5.

"A review of 21 studies about the effect of PA on mental health during the COVID-19 pandemic suggested that people who performed PA regularly with high volume and frequency and kept the PA routines stable would experience fewer symptoms of anxiety and depression [15]." (p.4)

"Although some studies have found that PA participation was positively associated with mental health among the Chinese adults during the COVID-19 pandemic [27,28], they did not specifically examine how the changes in PA and sitting time before and during the COVID-19 pandemic may affect mental health." (p.5)

Comments to the Author
Reviewer #1: PONE-D-21-27357

1. *Abstract*
   The aim of the study is to examine physical and sedentary behaviour changes and online behavior on mental health and life satisfaction – but isn't this regarding a specific time frame of lockdown during COVID (based on your title – "during the covid-19 lockdown")? The abstract does not state that this study is specifically looking at participants behavioural and psychological outcomes during lockdown. Please clarify.

   Response: Thanks for the comment. We have changed the title to "during the COVID-19 pandemic" since we share the same concern with the reviewer about the time frame of data collection. We have explained such a revision in response to the reviewer's comments #6 and #7. We stated explicitly that the study examined participants' behavioral and psychological outcomes during the COVID-19 pandemic in the revised Abstract.

   "This study aimed to investigate the effects of physical and sedentary behavioral changes and online behavior during the COVID-19 pandemic on mental health and life satisfaction among the Chinese population."

2. *Please clarify "first suffered from the COVID-19 pandemic between March 23 and April 9, 2020" - the introduction says COVID-19 was first reported in Dec 2019 and locked since January 23, 2020 and then relaxed March 23 and April 8 – How did they first suffer in March? Do you mean data was collected from March 23-April 9 regarding their experience with the lockdown from Jan-Mar 2020? Please clarify what March 23-April 9 is regarding as the article is about a lockdown period and this time frame is when lockdown was relaxed.*

   Response: Sorry for the confusion. We used "whose capital city Wuhan first suffered from the COVID-19 pandemic" to indicate that Hubei was the epicenter of the initial outbreak of the COVID-19 pandemic. To avoid confusion, we have deleted "whose capital city Wuhan first suffered from the COVID-19 pandemic" in the revised Abstract. The method section in the Abstract now reads:

   "The data were obtained from a cross-sectional survey of 2145 residents aged between 18 and 80 in Hubei province, China between March 23, 2020, and April 9, 2020."

3. *Introduction*
   Page 4 – you mention how the internet could enhance mental health (access to information, enabling people to work, study and shop online) – what about the ability for people to connect with loved ones/friends via online platforms (e.g., zoom, skype, facetime), especially during a lockdown. This would allow people to still "see" and talk to others during a lockdown. This could also help mental health. Also, gaming isn't necessarily a bad thing as people can game and chat with others which can be social, fun and something to do when in a lockdown. I'm not saying gaming could have negative effects, but it can also have good effects (specific to a lockdown).

   Response: Thanks for the comment. We have included such a variable in our study. Our measure of online communication refers to the contact with family and friends via online communication tools (e.g., WeChat). WeChat is the most widely used communication tool in China, enabling people to see and talk to others. Also, we agree with the reviewer that online entertainment, such as gaming, could be a stress coping strategy and positively affect mental

health during the pandemic. Our results actually support such an argument. The revised manuscript has included a hypothesis about Internet use and mental wellbeing. The corresponding changes are:

"(4) Internet use may be positively associated with mental wellbeing and life satisfaction during the COVID-19 pandemic." (p.6)

4. *Page 5 – "a study of 4,898 adolescents…" – this study is pre-COVID, correct?*

   Response: That study was conducted during the COVID-19 pandemic. We have clarified it in the revised manuscript.

   "A study of 4,898 adolescents in China showed that higher PA levels were significantly related to lower negative mood scores and higher positive mood scores during the COVID-19 pandemic" (p.5).

5. *The introduction doesn't emphasize that you are looking at the lockdown period of COVID. The article mentions "during the COVID-19 pandemic". Are you looking at the lockdown only or lockdown and non-lockdown periods during COVID? During a lockdown period, behavioural and psychological outcomes would be influenced more (can't leave their homes) vs non-lockdown periods since people can leave their homes. Please specific what time frame you are looking at and why this time frame (lockdown only or both lockdown and non-lockdown periods, etc.). Also, make sure you clearly state this time frame throughout the manuscript. Also, this seems to be looking at the first lockdown which is also important to state.*

   Response: Thanks for the comment. We would like to clarify the timeline of the lockdown of Hubei province. Chinese authorities imposed a lockdown on Wuhan, the capital city of Hubei province, on January 23, 2020, and enforced similar measures in 16 neighboring cities in Hubei province, affecting approximately 57 million people (Xiong, 2020). The authorities relaxed the Hubei lockdown on March 23, 2020, and officially lifted the Wuhan lockdown on April 8, 2020, after no new deaths transpired for the first time (He, 2020). The study was conducted between March 23, 2020, and April 9, 2020, when the lockdowns in Hubei were relaxed and eventually lifted. We asked the participants to report their physical activity participation and sitting time in the past two weeks and the past month, respectively, which may be during the lockdown or non-lockdown periods. Thus, we thought it would be better to use "COVID-19 pandemic" instead of "COVID-19 lockdown". We have used such a term throughout the manuscript.

6. *"the data collection was carried out between March 23 and April 9, 2020, while Hubei's lockdowns were gradually eased. Such data creates a unique opportunity for us to investigate Hubei residents' behavioral changes and psychological wellbeing during the lockdown period." – based on this sentence, my impression is you collected data during a non-lockdown period for participants to retrospectively report their behavioural and psychological outcomes during a lockdown (from Jan-Mar?), is this correct? You are specifically looking at lockdown effects. Why not collect data during the lockdown if its online anyways? Why wait until it eased when you are interested in lockdown effects? Please clarify.*

   Response: In the revised manuscript, we did not emphasize that we are looking at the lockdown period of the pandemic. We provided the reason in response to the reviewer's comment #5.

7. *Page 6 - Please specify the specific "online behaviors" you plan to examine in this study.*

   Response: Thanks for the suggestion. We have specified the "online behaviors" in the revised manuscript (p.6). The corresponding changes are:

   "Besides, we examined the impact of different types of Internet use, including information seeking, work/study, buying products, entertainment, reading/online learning, communication with family and friends, and investment/financial management."

8. *Methods*
   How did the participants get recruited? Any incentives for them to complete the survey?

   Response: We have provided more information about subject recruitment in the revised manuscript (p.7).

   "The online survey was carried out on a professional survey platform in China. The platform sent a notification to respondents in their sample bank with a link to allow access to the questionnaire. Only Hubei residents (with IP address locations in Hubei) can answer the questionnaire. No incentives were provided to the participants."

9. *Page 8 – for online behavior, what do you mean communication (talking with family members via zoom?). Please provide an example. Also, you looked at 7 internet uses which mostly seem like positive examples (based on your introduction) – you mention not having a hypothesis, but you only looked at internet uses that could enhance mental health; thus I would hypothesis internet use will have positive effects, correct? That said, why not include the negative internet uses you mentioned in the introduction such as gambling, social media use, shopping? Also, im assuming "buying products" is referring to "shop online that regularizes their routines". Please provide an example of buying products.*

   Response: Thanks for the questions. To clarify, communication in this study was measured by asking the respondents whether they communicated with family and friends via online communication tools (e.g., WeChat). WeChat is the most widely used communication app in China, which acted as critical infrastructure in fulfilling people's practical, emotional, and medical needs during the COVID lockdown in China (Qian & Hanser, 2020). We have clarified this in the revised manuscript as below.

   "communication with family and friends via online communication tools (e.g., WeChat)" (p.9).

   As the reviewer suggested, we have included an example for buying products in the revised manuscript as "buying products (e.g., groceries)" (p. 9).

10. *Page 8 – Did you explain what "moderate to vigorous physical activity" means to the participant in the survey (e.g., moderate to vigorous physical activity means any activity that your heart rate increases and you are sweating) or leave it to the participant to interpret it? For example, someone may interpret physical activity as anything active (e.g., doing daily chores) within a day while someone else may interpret specific working out activities (e.g., running for an hour). As you can imagine, a daily chore may not be as "active" as someone who runs for an hour. You mention in the discussion that future studies should look at type of physical activity, which I agree is very important, but curious how much information was given to the participants regarding this question.*

Response: We asked the participants about the frequency of doing moderate-to-vigorous physical activity (MVPA), followed by a brief explanation and some examples of MVPA. In the survey, MVPA refers to exercises such as running, playing ball, swimming, dancing, brisk walking, etc. for ≥10 minutes. Typically, physical activity needs to continue for at least 10 minutes to be considered a session of exercise. We have clarified the measure of "moderate to vigorous physical activity" in the revised manuscript as below:

"Participants reported the frequency of doing moderate-to-vigorous physical activity (MVPA) in 2019 and in the past month, followed by a brief explanation and some examples of MVPA. MVPA refers to exercises such as running, playing ball, swimming, dancing, brisk walking, etc., for ≥10 minutes, which cause breathing and heart rate to increase." (p.8)

11. *Page 8 – "information seeking" – is this specific COVID information seeking? Or other types of information seeking? How did you word this question?*

Response: Thanks for the question. The information seeking was measured by general information seeking behavior, not specific COVID-19 information seeking. We have clarified this in the revised manuscript (p.9).

"Seven main and conceptually different Internet uses were accessed, including general information seeking;"

12. *Results*
You have most of the information from Table 1 and 2 in text (e.g., "half of the respondents were female (51.28%)."). Thus, Tables 1 and 2 are redundant. I would put all the information in text and remove Tables 1 and 2. Please include the sample size for each variable (e.g., half of the respondents were female (n = X; 51.28%)). Or keep Table 1 and 2 but remove the text because it is redundant. If you keep the tables – add the sample size for each variable and separate the Mean (SD) data into one column and the % data into another column.

Response: Thanks for the suggestions. To be consistent with the reporting style in most studies, we decided to keep Tables 1 and 2. As the reviewer suggested, we have removed the statistics and only kept a very brief description of PA, sitting time and online behavior in the revised manuscript.

13. *Page 10 - The "M =" are missing for online class, work and study, buying products, investment and financial management.*

Response: Thanks for pointing this out. However, as the reviewer suggested in Comment #12, we have deleted these statistics as they are redundant.

14. *Regression results – since you have table 3 with all the test statistics, you don't need to have the statistics in text. You can remove the test statistic information and just explain the findings, as you have done (i.e., remove the numbers from this section that are in Table 3).*

Response: Thanks for the suggestion. We have removed the statistics in the text.

15. *Most participants completed the survey alone online, but some participants completed via phone survey. Any differences between the style of data collection (e.g., participants could ask for clarification during a phone survey from the research assistant vs participants who*

completed the survey alone on a computer). Were research assistants asked to not provide any additional information but simply read the questions to the participants over the phone? How many completed the survey via phone vs online?

Response: Thanks for the questions. We have clarified the two modes of data collection in the revised manuscript as below:

"There are minimal differences between the two modes of data collection. Our research staff will be asked not to provide any additional information but simply read the questions to the participants." (p.7)

We also included the number of participants who completed the survey online or via telephone in the revised manuscript.

"After deleting cases with at least one missing value, the final sample included 2145 respondents, of which 1944 (90.63%) completed the online survey, and 201 (9.37%) completed the phone survey." (p.7)

16. *Discussion*
    Page 13 – I would not assume communication referring to use of social media, unless the question was framed this way. I would assume communication meaning communicating with others via phone or zoom/skype etc. When you clarify what "communication" means in the methods, this may make sense though.

    Response: Thanks for the question. We have clarified this measurement in the revised manuscript.

    "communication with family and friends via online communication tools (e.g., WeChat)"

17. *I believe it is important to state that this study is looking at a lockdown period which is very different from normal day to day life (pre- and post-COVID). Something that was good during a lockdown (e.g., can't work so they play video games all day for social interaction and something to do), may not be good when the lockdown is lifted (e.g., playing video games but should be at work). Thus, these findings are only relevant when a city/town are under lockdown.*

    Response: Thanks again for the comment. As we responded to the reviewer earlier, we would not emphasize that we are looking at the lockdown effects. We use "during the COVID-19 pandemic" throughout the manuscript.

18. *Page 13 - please clarify what shopping means? Shopping for non-essentials (e.g., equipment) vs essentials (e.g., food)? Or does online shopping have a negative effect even when shopping for essentials (i.e., any type of shopping)? Though, I thought online shopping for essentials could give the impression of regularize daily routines which would be good for mental health? Please clarify.*

    Response: Thanks for the comment. We did not specify types of shopping in the survey. It could be shopping for essentials or non-essentials.

19. *Page 14 – another potential limitation – participants had to retrospectively report their physical activity and sitting time prior to COVID (especially 2019 MVPA rates) which could be inaccurate since it was based on memory (especially among older adults).*

Response: Thanks for pointing this out. We have added the reporting bias in the limitation of the revised manuscript. The corresponding revisions are shown below:

"Moreover, we asked participants to retrospectively report their physical activity and sitting time prior to the COVID-19 pandemic, which may be subject to recall bias due to inaccurate memory. Future studies may use objective or direct measures of physical activity to increase precision and validate the self-report measures." (p.15)

References:

He J. China's State Machinery Will Beat Coronavirus Crisis, but at What Cost? South China Morning Post, February 1, 2020.

Xiong, Y. All major cities in China's Hubei province under lockdown. 2020. Retrieved from. https://edition.cnn.com/asia/live-news/coronavirus-outbreak-02-02-20-intl-hnk/h_9d7932db226e2e5109eabc8e4f5ae6c9

Qian Y, Hanser A. How did Wuhan residents cope with a 76-day lockdown?. Chinese Sociological Review. 2021 January 1;53(1):55-86.
